# Didemnosides A and B: Antiproliferative Nucleosides from the Red Sea Marine Tunicate *Didemnum* Species

**DOI:** 10.3390/md23070262

**Published:** 2025-06-23

**Authors:** Lamiaa A. Shaala, Diaa T. A. Youssef, Hadeel Almagthali, Ameen M. Almohammadi, Wafaa T. Arab, Torki Alzughaibi, Noor M. Bataweel, Reham S. Ibrahim

**Affiliations:** 1 Suez Canal University Hospitals, Suez Canal University, Ismailia 41522, Egypt; 2 Department of Natural Products, Faculty of Pharmacy, King Abdulaziz University, Jeddah 21589, Saudi Arabia; 3 King Fahd Medical Research Center, King Abdulaziz University, Jeddah 21589, Saudi Arabia; taalzughaibi@kau.edu.sa; 4 Department of Pharmacognosy, College of Pharmacy, Taif University, Al-Haweiah 21974, Saudi Arabia; hadeel.g@tu.edu.sa; 5 Department of Pharmacy Practice, Faculty of Pharmacy, King Abdulaziz University, Jeddah 21589, Saudi Arabia; amalmohammadi@kau.edu.sa; 6 Vaccines and Immunotherapy Unit, King Fahd Medical Research Center, King Abdulaziz University, Jeddah 21589, Saudi Arabia; wtarab@kau.edu.sa; 7 Department of Medical Laboratory Sciences, Faculty of Applied Medical Sciences, King Abdulaziz University, Jeddah 21589, Saudi Arabia; 8 Department of Biological Science, Faculty of Science, King Abdulaziz University, Jeddah 21589, Saudi Arabia; no0ora.118@hotmail.com; 9 Department of Pharmacognosy, Faculty of Pharmacy, Alexandria University, Alexandria 21521, Egypt; reham.abdelkader@alexu.edu.eg

**Keywords:** Red Sea tunicate, *Didemnum* species, bioactive compounds, didemnosides A and B, human cancer cell lines, antiproliferative activity, molecular docking

## Abstract

Marine tunicates are a very attractive and abundant source of secondary metabolites with chemical diversity and biological activity. Fractionation and purification of the organic extract of the Red Sea tunicate *Didemnum* species resulted in the isolation and identification of three new compounds, didemnosides A and B (**1** and **2**) and 1,1′,3,3′-bisuracil (**3**), together with thymidine (**4**), 2′-deoxyuridine (**5**), homarine (**6**), and acetamide (**7**). Planar structures of the compounds were explained through analyses of their 1D (^1^H and ^13^C) and 2D (^1^H–^1^H COSY, HSQC, and HMBC) NMR spectra and high-resolution mass spectral determinations. Compound **1** exhibited the highest growth inhibition toward the MCF-7 cancer cell line with IC_50_ values of 0.597 μM, while other compounds were inactive (≥50 μM) against this cell line. On the other hand, compounds **1**, **2**, and **4**–**7** moderately inhibited SW-1222 and PC-3 cells with IC_50_ values ranging between 5.25 and 9.36 μM. Molecular docking analyses of the top three active compounds on each tested cell line exposed stable interactions into the active pockets of estrogen receptor alpha (ESR1), human topoisomerase II alpha (TOP2A), and cyclin-dependent kinase 5 (CDK5) which are contemplated as essential targets in cancer treatments. Thus, compound **1** represents a scaffold for the development of more effective anticancer drugs.

## 1. Introduction

The Red Sea is widely recognized as one of the world’s foremost biodiversity hotspots, boasting an exceptionally high rate of endemism compared to adjacent marine regions [1]. This remarkable endemism is likely due to the Red Sea’s partial isolation, with the Gulf of Aden strait of Bab al-Mandab in the south acting as a natural barrier. Environmental factors such as temperature, salinity, and shallow depths restrict the movement of species between the Red Sea and the Indian Ocean [2]. To the north, the Red Sea is linked to the Mediterranean Sea via the Suez Canal, enabling a northward, unidirectional flow of species, a phenomenon known as Lessepsian migration [3]. Additionally, the Red Sea is characterized by its warm, saline deep waters, which are nearly isothermal and isohaline (around 22 °C and 40‰) across large portions of its depths [4]. These conditions support a highly specialized and often endemic fauna. The Red Sea’s biodiversity is further enriched by its expansive coral reefs, which span more than 16,000 km^2^ [5]. Given these unique features and the fact that estimating the full diversity of species remains challenging due to the limited number of studies, the Red Sea represents a captivating area for scientific exploration of its chemical biodiversity and biomedical importance.

Marine tunicates are well known as one of the richest sources of diverse bioactive natural products. Like many other invertebrates, tunicates rely on chemical defenses for survival. Consequently, these chemicals have been evaluated for their potential as drug leads and candidates [6]. Tunicates host a variety of bacterial symbionts, which play a key role in the production of many of the defensive chemicals found in these organisms [6]. The wide array of natural products obtained from tunicates is largely attributed to their consistent association with symbiotic bacteria [7,8,9]. These natural products exhibit a broad spectrum of bioactivities, including antitumor [10,11,12,13,14,15,16], antiviral [17], antibacterial [18], and many others [19]. Remarkably, tunicates have provided three marketed anticancer drugs including trabectedin (ET-743) (Yondelis^®^), plitidepsin (Aplidin^®^), and lurbinectedin (Zepzelca™) [20]. Additionally, marine tunicates are considered promising sources of natural antibiotics. The family Didemnidae, which includes the genus *Didemnum*, is one of the largest families within the tunicate group [21]. The genus *Didemnum* has been described more frequently than other genera within the Didemnidae family [22]. Recognized as one of the most intriguing sources of bioactive secondary metabolites among tunicates, *Didemnum* hosts a variety of symbiotic bacteria, some of which are responsible for the production of these metabolites [23]. These metabolites demonstrate a wide range of bioactivities, including antitumor, antiviral, antibacterial, and others [19].

Chemical investigation of the Red Sea tunicate *Didemnum* species led to the isolation of three new compounds named didemnosides A and B (**1** and **2**), and 1,1′,3,3′-bisuracil (**3**), along with the previously reported compounds thymidine (**4**) [24], 2′-deoxyuridine (**5**) [25,26], homarine (**6**) [27,28], and acetamide (**7**). In this study, we report the purification, structural determination as well as the evaluation of the antiproliferation effects of the compounds. Additionally, computational studies were conducted on the most active compounds to explore the molecular targets associated with the tested cancerous cells.

## 2. Results and Discussion

### 2.1. Purification of Compounds ***1***–***7***

**Figure 1:**
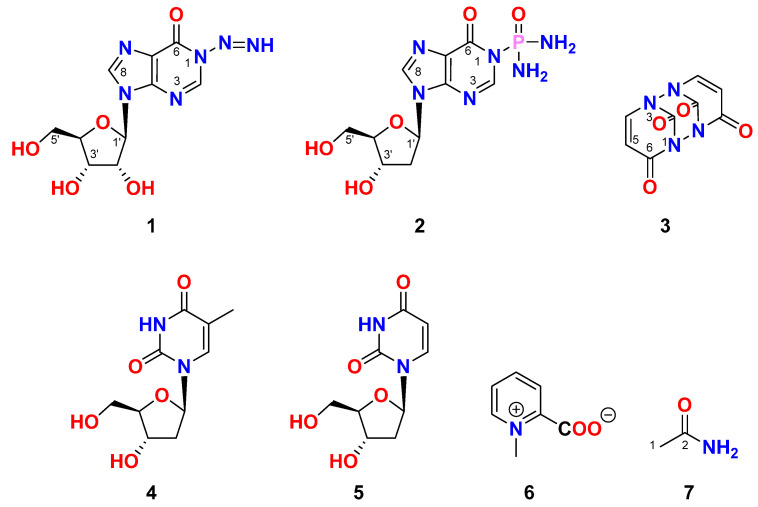
Chemical structures of compounds **1**–**7**.

The freeze-dried tunicate Didemnum species was extracted three times with a mixture of CH_2_Cl_2_-CH_3_OH (1:1) at room temperature. The combined organic extracts were subjected to chromatographic partition and fractionations on normal and reversed-phase silica columns and final HPLC purification to give seven compounds (**1**–**7**) (Figure 1).

### 2.2. Structure of Compound ***1***

Compound **1** (Figure 1) possesses the molecular formula C_10_H_12_N_6_O_5_ as supported by the pseudomolecular ion peak at *m/z* 319.0766 [M + Na]^+^ in the (+)-HRESIMS spectrum (Figure S1). The ^1^H and ^13^C NMR spectra (Figures S2 and S3) of compound **1** (Table 1) showed characteristic signals for inosine [25]. Those signals at δ_H/C_ 8.05 (1H, s)/145.9 (CH, C-2), 148.2 (C, C-4), 124.4 (C, C-5), 156.6 (C, C-6), and 8.31 (1H, s)/138.7 (CH, C-8) are characteristic for the purin-6-one part (Part **A**) of inosine [25]. The remaining signals at δ_H/C_ 5.85 (1H, d, *J* = 5.7 Hz)/87.4 (CH, C-1′), 4.46 (1H, t, *J* = 5.2 Hz)/74.1 (CH, C-2′), 5.52 (brs, O*H*-2′), 4.11 (1H, dd, *J* = 4.8, 3.8 Hz)/70.3 (CH, C-3′), 5.25 (brs, O*H*-3′), 3.92 (1H, q, *J* = 3.8 Hz)/85.6 (CH, C-4′), 3.64 (1H, dd, *J* = 12.0, 4.0 Hz), 3.54 (1H, dd, *J* = 12.0, 4.0 Hz)/61.3 (CH_2_, C-5′) and 5.08 (brs, O*H*-5′) are characteristic for a ribose moiety (Part **B**) [25]. Further, a non-interrupting coupling system was traced from H-1′ to H_2_-5′ in the COSY experiment (Figure S4), confirming this assignment of the ribose moiety.

**Figure 2:**
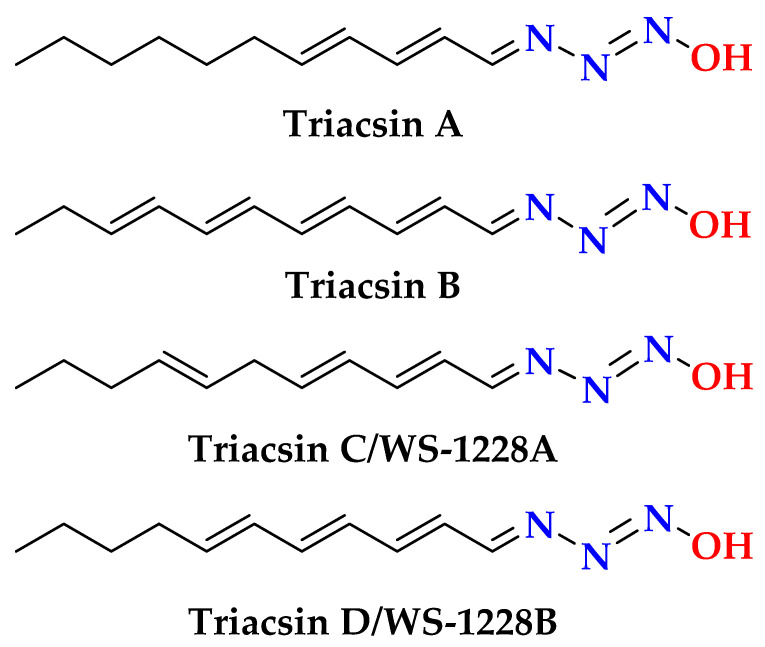
Selected examples of natural products with *N*-diazenyl moiety [29].

The protons-bearing carbons of **1** were assigned from the HSQC experiment (Figure S5). Moreover, the linkage of the ribose moiety with the purin-6-one part through C-1’ and N-7 was supported by the HMBC correlations (Figure S6) between H-1’ (δ_H_ = 5.85) and the signal at δ_C_ 148.2 (C-4) and 138.7 (C-8) as well as between H-8 (δ_H_ = 8.31) and the signal at δ_C_ 87.4 (C-1’). The sum of the molecular elements of parts **A** and **B** is C_10_H_11_N_4_O_5_. The absence of the expected and significant downfield signal of the amidic proton (N*H*-1) between 10 and 16 ppm in the ^1^H NMR spectrum acquired in DMSO-*d*_6_ [25] supported the absence of this proton. The remaining elements of N_2_H along with the absence of the amidic proton (N*H*-1) [25] support the placement of the remaining elements (N=NH) at *N*-1 of the purine moiety to complete the molecular formula of **1**. The existence of naturally occurring compounds with diazenyl (N=NH) and *N*-diazenyl (N-N=NH) moieties is well documented in the literature [29]. Selected examples of such compounds include triacsins A and B from *Streptomyces* sp. and triacsins C and D isolated from *Streptomyces aureofaciens* [29] are outlined in Figure 2.

**Figure 3:**
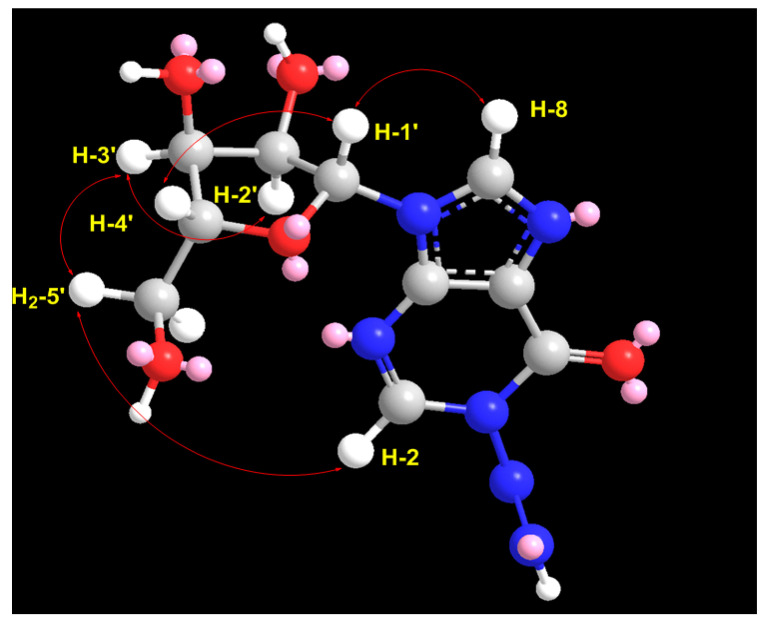
Significant observed ^1^H-^1^H ROESY correlations of compound **1**.

In the ^1^H–^1^H ROESY spectrum of compound **1**, a strong correlation between H-1′ (δ_H_ = 5.85) and H-4′ (δ_H_ = 3.93), along with a correlation between H-3′ (δ_H_ = 4.11) and H-4′ (δ_H_ = 3.92), supports the β-configuration of the ribose moiety (Table 1; Figure 3 and Figure S7). Additional expected ROESY correlations for compound **1** are also presented in Table 1; Figure 3 and Figure S7. Furthermore, the optical rotation of compound 1, [α]_D_ = −55° (c 0.1, MeOH), is consistent in both sign and magnitude with that of inosine ([α]_D_ = −52°, c 1.0, H_2_O) [30], further supporting the presence of a d-ribose moiety. Based on these spectroscopic and chiroptical data, compound **1** was identified as 1-diazenyl-9-((2*R*,3*R*,4*S*,5*R*)-3,4-dihydroxy-5-(hydroxymethyl)tetrahydrofuran-2-yl)-1,9-dihydro-6H-purin-6-one (*N*-diazenylinosine), with the SMILES notation: O=C1C2=C(N([C@H]3[C@H](O)[C@H](O)[C@@H](CO)O3)C=N2)N=CN1N=N. This compound is considered a novel natural product and has been named didemnoside A.

### 2.3. Structure of Compound ***2***

Compound **2** (Figure 1) has a molecular formula of C_10_H_15_N_6_O_5_P, as supported by the pseudomolecular ion peak at *m*/*z* 353.0738 [M + Na]^+^ in the (+)-HRESIMS spectrum (Figure S8). Like **1**, the absence of any significant downfield signals in the ^1^H NMR spectrum of **2** between 10 and 16 ppm in the DMSO-*d_6_* proves again the absence of the proton at N-1 and its substitution. The ^1^H NMR and ^13^C NMR data of **2** (Table 2 and Figures S9 and S10) are like those of **1** except for the substituents at the N-1 of the purin-6-one part and the existence of 2′-deoxyribose moiety instead of 2′-ribose in **1**. The ^1^H/^13^C NMR signals at δ_H/C_ 8.04 (1H, s)/146.2 (CH, C-2), 147.9 (C, C-4), 124.4 (C, C-5), 157.1 (C, C-6), and 8.27 (1H, s)/138.3 (CH, C-8) are characteristic for the purin-6-one part of the molecule [25]. Further signals at δ_H/C_ 6.30 (dd, *J* = 7.2, 6.3 Hz)/83.6 (CH, C-1′) 2.63 (1H, ddd, *J* = 13.3, 7.5, 5.8 Hz), 2.28 (1H, ddd, *J* = 13.3, 6.2, 3.2 Hz)/39.9 (CH_2_, C-2′), 4.38 (quin., *J* = 3.0 Hz)/70.6 (CH, C-3′), 5.34 (brs, O*H*-3′), 3.86 (q, *J* = 4.4 Hz)/87.9 (CH, C-4′), 3.59 (1H, dd, *J* = 11.8, 4.6 Hz), 3.51 (1H, dd, *J* = 11.8, 4.4 Hz)/61.6 (CH_2_, C-5′), and 5.08 (brs, O*H*-5′) are characteristic for 2′-deoxyribose moiety [25]. Further, the COSY experiment (Figure S11) confirmed the existence of a single continuous spin-coupling system from the methylne protons at C-1′ to the methylene protons at C-5′ within the deoxyribose unit of **2** confirming this assignment. Also, the assignment of the proton-bearing carbons was assigned from the multiplicity-edited HSQC experiment (Figure S12).

**Figure 4:**
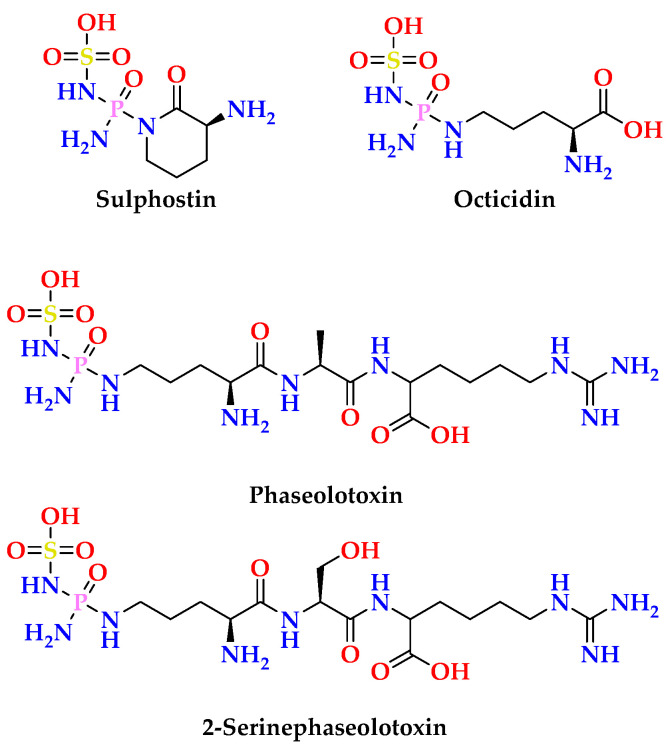
Selected examples of natural products with *N-*phosphonic acid diamide moiety [31].

Moreover, the linkage of the ribose moiety with the purin-6-one part through C-1′ and N-7 was supported by the HMBC correlations (Figure S13) between H-1′ (δ_H_ = 6.30) and the signal at δ_C_ 147.9 (C-4) and 138.3 (C-8) as well as between H-8 (δ_H_ = 8.27) and the signal at δ_C_ 83.6 (C-1′). Similarly, the sum of the molecular elements of subunits **A** and **B** in **2** is C_10_H_11_N_4_O_4_. The remaining elements of PON_2_H_4_ (phosphonic acid diamide moiety) along with the absence of the amidic N*H* in the ^1^H NMR spectrum of **2** recorded in DMSO-*d*_6_ place this remaining moiety at N-1. The existence of *N-*phosphonic acid diamide moiety is a common structural motif in many naturally occurring secondary metabolites [31]. Some of the reported natural products with *N-*phosphonic acid diamide moiety are outlined in Figure 4.

**Figure 5:**
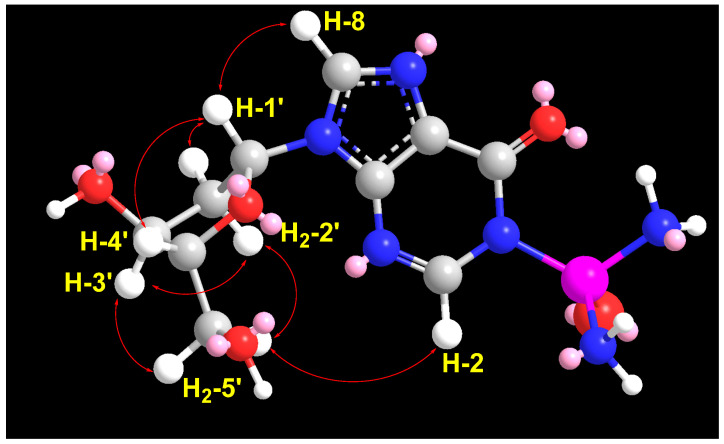
Significant observed ^1^H-^1^H ROESY correlations of compound **2**.

Similarly, a significant ROESY correlation between protons H-1′ (δ_H_ = 6.30) and H-4′ (δ_H_ = 3.86) (Figure 5 and Figure S14), combined with the absence of ROESY correlations between H-3′ (δ_H_ = 4.38) and H-4′ (δ_H_ = 3.86) as well as between H-1′ and H-3′, confirms the β-configuration of the 2′-deoxyribose moiety (Table 2 and Figure 5). Additional expected ROESY correlations observed for compound **2** are detailed in Table 2 and Figure 5 and Figure S14. Furthermore, the optical rotation of compound **2**, [α]_D_ = −26° (c 0.1, MeOH), closely matches both the sign and magnitude of the reported values for 2′-deoxyinosine ([α]_D_ = −22 to −17°, c 1.0, H_2_O) [32], supporting the presence of a d-2′-deoxyribose moiety in compound **2**. Based on these findings, compound **2** was identified as P-(9-((2*R*,4*S*,5*R*)-4-hydroxy-5-(hydroxymethyl)tetrahydrofuran-2-yl)-6-oxo-6,9-dihydro-1H-purin-1-yl)phosphinic diamide (2′-deoxyinosine-1-phosphonic acid diamide) with the SMILES notation: NP(=O)(N)N1C=Nc2c(ncn2[C@H]3C[C@H](O)[C@@H](CO)O3)C1=O. This compound is considered a new natural product and has been named didemnoside B.

### 2.4. Structure of Compound ***3***

Compound **3** (Figure 1) was determined to have the molecular formula C_8_H_4_N_4_O_4_, as confirmed by the ion peak at *m*/*z* 243.0127 [M + Na]^+^ observed in the (+)-HRESIMS spectrum (Figure S15). The ^1^H and ^13^C NMR spectra of compound **3** (Figures S16 and S17) displayed chemical shifts consistent with those reported for uracil [33], including signals at δ_H/C_ 151.5 (C, C-2), 164.3 (C, C-4), 5.45 (d, *J* = 7.6 Hz)/100.2 (CH, C-5), and 7.39 (d, *J* = 7.6 Hz)/142.3 (CH, C-6) (Table 3). Notably, the expected downfield ^1^H NMR signals corresponding to the amidic protons N*H*-1 and N*H*-3, which typically appear between 10 and 16 ppm in DMSO-*d*_6_, were absent. Additionally, the ^13^C NMR spectrum exhibited only four distinct carbon resonances. Together, these observations strongly suggest a symmetric dimeric structure, likely formed via N-1/N-1′ and N-3/N-3′ linkages. A structurally similar compound, 1,1′-biuracil, was previously isolated from *Epidermidibacterium keratini* EPI-7 [34]. Based on the spectral data and structural considerations, compound **3** was identified as 1,2,6,7-tetraazatricyclo [5.3.1.1^2,6^]dodeca-4,8-diene-3,10,11,12-tetraone, representing a new natural product, herein named 1,1′,3,3′-biuracil.

### 2.5. Structure of Compound ***4***

Compound **4** (Figure 1) displayed the molecular formula C_10_H_13_N_2_O_5_ as supported by the pseudomolecular ion peak at *m*/*z* 243.1 [M + H]^+^ in the ESIMS spectrum. Its ^1^H and ^13^C NMR data are like those reported for thymidine [35,36,37]. Accordingly, **4** was assigned as thymidine.

### 2.6. Structure of Compound ***5***

Compound **5** (Figure 1) has a molecular formula C_9_H_12_N_2_O_5_ as supported by the ion peak at 229.08 [M + H] ^+^ in the ESIMS spectrum. The ^1^H and ^13^C NMR spectra are like those reported for 2′-deoxyuridine [26,36,37]. Thus, **5** was assigned as 2′-deoxyuridine.

### 2.7. Structure of Compound ***6***

Compound **6** (Figure 1) with a chemical formula of C_7_H_7_O_2_N, as established from the ESIIMS ion peak at *m*/*z* 138.1 [M + H]^+^. The ^1^H and ^13^C NMR data of compound **6** are like those reported for homarine [27,28]. Thus, **6** was assigned as homarine. Nevertheless, this is the earliest record of the existence of this compound in the genus *Didemnum*.

### 2.8. Structure of Compound ***7***

Compound **7** (Figure 1) was assigned the molecular formula C_2_H_5_NO, as evidenced by the ion peak at *m*/*z* 60.0 [M + H]^+^ in the ESIMS spectrum (Figure S18). The ^1^H and ^13^C NMR spectra (Figures S19 and S20) revealed signals at δ_H/C_ 1.75 (3H, s)/22.5 (CH_3_, C-1), 171.6 (C, C-2) (Table 4), along with broad singlets at δ_H_ 6.68 and 7.40 ppm corresponding to the amide protons (NH_2_) at C-1. These spectral features are consistent with those reported for synthetic acetamide [38]. Accordingly, compound **7** was identified as acetamide. To the best of our knowledge, this represents the first report of acetamide being isolated from a marine natural source.

### 2.9. Antiproliferative Activities of the Compounds

The cytotoxic activities of the compounds were evaluated using the MTT assay against three human cancer cell lines: colorectal cancer (SW-1222), breast cancer (MCF-7), and prostate cancer (PC-3). Among the tested compounds, compound **1** exhibited the most potent activity, showing selective cytotoxicity against MCF-7 cells with an IC_50_ value of 0.597 μM, whereas the other compounds were inactive against this cell line (IC_50_ ≥ 50 μM) (Table 5). In contrast, all compounds demonstrated moderate activity against SW-1222 and PC-3 cell lines, with IC_50_ values ranging from 5.25 to 7.07 μM and 7.16 to 9.36 μM, respectively. These findings highlight the potential significance of the *N*-diazenyl moiety at N-1 in conferring selective anticancer activity, particularly against breast cancer cells. Moreover, the results underscore the value of nucleoside derivatization as a strategic approach for developing novel anticancer agents with improved selectivity and potency.

### 2.10. Antimicrobial Activities of the Compounds

The antimicrobial activity of compounds **1**–**7** was evaluated using the disc diffusion assay at concentrations of 50 μg/disc against a range of pathogenic microorganisms, including methicillin-resistant *S. aureus* (MRSA) (ATCC^®^ 33591), *S. aureus* (ATCC^®^ 12600), *P. aeruginosa* (ATCC^®^ 9027), and *E. coli* (ATCC^®^ 11775). However, none of the compounds demonstrated any significant antimicrobial activity against the tested pathogens.

### 2.11. Computational Studies on Molecular Targets Associated with Tested Cancerous Cells

Molecular docking analysis of the top three active compounds on each tested cell line exposed stable interactions into the active pockets of estrogen receptor alpha (ESR1), human topoisomerase II alpha (TOP2A), and cyclin-dependent kinase 5 (CDK5) which are contemplated as essential targets in cancer treatments

**Figure 6:**
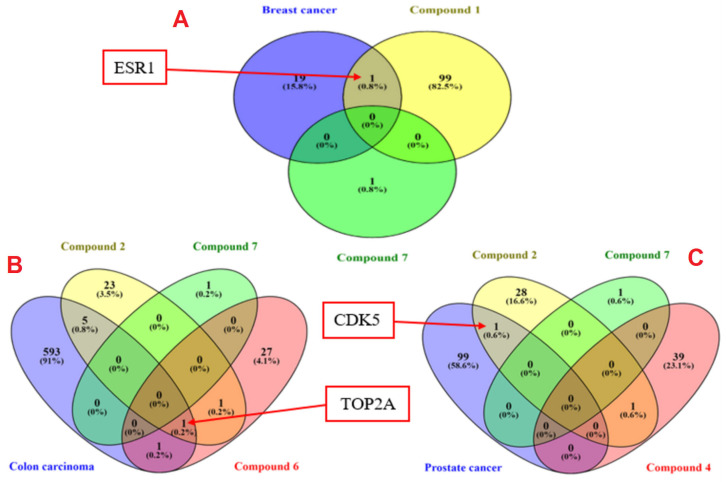
Venn diagrams for common molecular targets between compounds **1**, **7**, and human breast cancer (**A**)**,** compounds **2**, **6**, **7,** and colon cancer (**B**), and between compounds **2**, **4**, **7,** and prostate cancer (**C**).

The criteria for selecting ESR1, TOP2A, and CDK5 for further in silico studies was demonstrated in Figure 6. The genes related to the top three antiproliferative compounds on each cell line were separately predicted using the SwissTargetPrediction database. Whereas human GeneCards database was utilized for mining the targets related to each cancer type namely, colorectal cancer cells (SW-1222), breast cancer cells (MCF-7), and prostate cancer (PC-3). Figure 6 depicts that ESR1 is the common target between breast cancer and compounds **1** and **7**. The common gene between compounds **2**, **6,** and **7** with colon carcinoma was TOP2A. Finally, CDK5 was common between compounds **2**, **4,** and **7** with prostate cancer. For this reason, compound **1** was promoted for further molecular docking study by Glide extra-precision (Xp) module against ESR1 (PDB ID 6CBZ), and compound **2** against TOP2A (5GWK) and CDK5 (1UNH) to explicate their binding modes at molecular levels.

#### 2.11.1. Molecular Docking with ESR1

**Figure 7:**
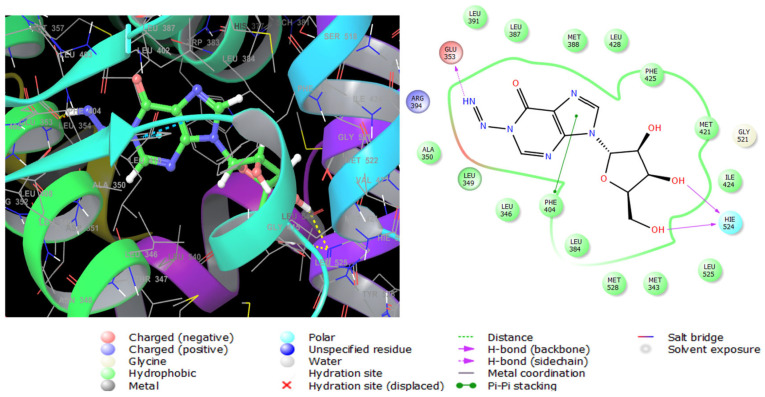
Three-dimensional (**left**) and two-dimensional (**right**) interaction diagrams of compound **1** with the crystal structure of ESR1 (PDB ID: 6CBZ).

Estrogen performs its biological action via binding to alpha- and beta-estrogen receptors, which belong to the nuclear receptor transcription factor superfamily and have highly conserved DNA- and ligand-binding domains [39]. These nuclear receptors play an important role in gene regulation. When this gene is mutated, it creates an aberrant version of the estrogen receptor, generally known as ER+, whose expression causes breast cancer [40]. Compound **1** exerted potent antiproliferative activity on human estrogen receptor-positive breast cancer cells (MCF-7), followed by compound **7** (Table 6). Therefore, these two compounds were selected for glide extra-precision docking protocol to unravel the binding mechanisms with ESR1 (6CBZ) in a complex with estradiol. The docking scores of compounds **1** and **7** are depicted in Table 6, where the first compound showed low binding energy with an XP Gscore of -7.700 Kcal mol^−1^. The selective antiproliferative effect of compound **1** was further confirmed by molecular docking analysis, where the XPG scores of compound **1** on ESR1 (identified gene associated with human breast cancer), TOP2A (identified gene associated with colon carcinoma), and CDK5 (identified gene associated with prostate cancer) were −7.700, ≥−5.000, and ≥−5.000, respectively. The docking scores in Table 6 reflected the most stable binding of compound **1** with ESR1. Therefore, compound **1** was promoted for in-depth molecular docking analysis on ESR1 to investigate the underlying molecular interactions in the receptor active site). The 2D and 3D molecular interactions of compound **1** with ESR1 are shown in Figure 7. It was found to be engaged by three hydrogen bonds between imino, hydroxyl groups, and Glu353, Hie524, respectively, in the receptor active site. Pi–Pi stacking interaction was also observed between the imidazole ring and Phe404. Moreover, it interacts hydrophobically with numerous amino acid residues such as Leu3, Met421, Ile424, and Phe425. These interactions were very close to those observed for estradiol; the native ligand of ESR1 as shown in Figure S21. In addition, the observed interactions were also like ESR1 inhibitors reported in the literature [41], certifying the successful docking in the right receptor pocket.

#### 2.11.2. Molecular Docking with TOP2A

**Figure 8:**
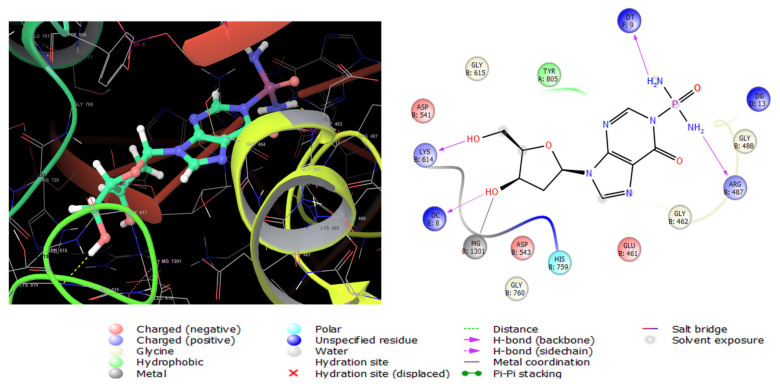
Three-dimensional (**left**) and two-dimensional (**right**) interaction diagrams of compound **2** with the crystal structure of TOP2A (PDB ID: 5GWK).

Topoisomerase II alpha (TOP2A) is a nuclear enzyme that regulates the DNA tertiary structure. The overexpression of (TOP2A) is highly associated with tumor behavior alterations and chemotherapeutic resistance in colon cancer [42]. Compounds **2**, **7,** then **6,** showed high antiproliferative activities when tested in vitro against SW 1222 colon carcinoma cells (Table 5). For this sake, the three compounds were subjected to docking analysis using the extra precision protocol on the crystal structure of the TOP2A (5GWK) complexed with the known anticancer etoposide. Compound **2** showed the lowest binding score (Table 6) conforming with in vitro results. The molecular interactions of compound **2** with the enzyme are displayed in Figure 8. It interacts with Lys614 andDc8 with two hydrogen bonds by its two hydroxyl groups, in addition to another two hydrogen bonds between the two amino groups with Arg487 as etoposide (Figure S22) and Dt9. Moreover, a metal coordinate bond with magnesium 1301 was formed, and a hydrophobic interaction with Tyr805.

#### 2.11.3. Molecular Docking with CDK5

**Figure 9:**
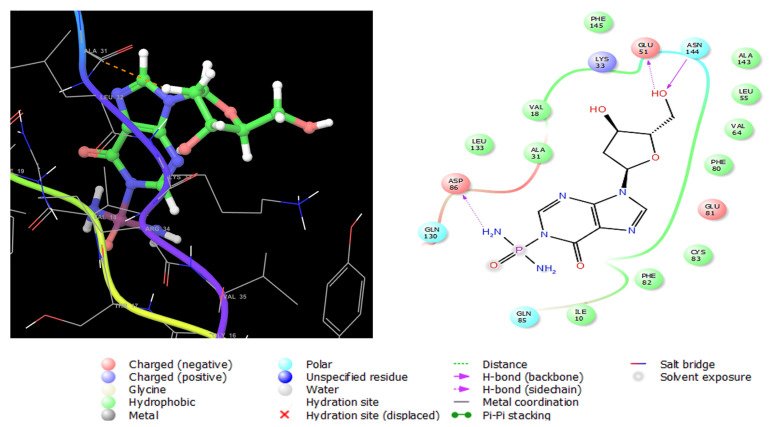
Three-dimensional (**left**) and two-dimensional (**right**) interaction diagrams of compound **2** with the crystal structure of CDK5 (PDB ID: 1UNH).

Cyclin-dependent kinase 5 (CDK5) is a serine/threonine kinase structurally like other CDKs [43]. It is a promising target for the treatment of prostate cancer; this enzyme is essential in the growth of prostate tumors and metastases [44]. Like ESR1 and TOP2A, compounds **2**, **4**, and **7**, having lower IC_50_ values on human prostate cancer (PC-3) cells (Table 5), were docked on CDK5 enzyme (PDB ID: 1UNH) co-crystallized with indirubin-3′-monoxime as an inhibitor (Figure S23). The glide XP Gscores of the three compounds are presented in Table 6. Interactions of compound **2** with CDK5 (1UNH) are shown in Figure 9, where it was anchored to the enzyme’s active sites with three hydrogen bonds with Glu51, Gln130, and Asn144. Moreover, a negatively charged interaction with Asp86 was formed, and polar engagement with Gln85, Gln130, and Asn144. Numerous van der Waals hydrophobic interactions were formed with Ile10, Val18, Ala31, Leu 55, Val64, Phe80, Phe82, and Cys83. These diverse interactions accounted for the stable conformation of compound **2** in the active site of CDK5.

## 3. Materials and Methods

### 3.1. General Experimental Procedures

Optical rotations were acquired on a digital DIP-370 polarimeter (JASCO, Oklahoma City, OK, USA). One- and two-dimensional NMR spectra were acquired on Bruker Avance DRX 600 MHz spectrometer using DMSO-*d*_6_ as solvent. NMR spectra were referenced to the residual protonated solvent signals (DMSO-*d*_6_: 2.50 ppm for ^1^H and 39.51 ppm for ^13^C. Positive ion HRESIMS data were obtained with a Micromass Q-ToF equipped with leucine enkaphalin lockspray, using *m*/*z* 556.2771 [M + H]^+^ as a reference mass.

### 3.2. The Red Sea Didemnum Species

**Figure 10:**
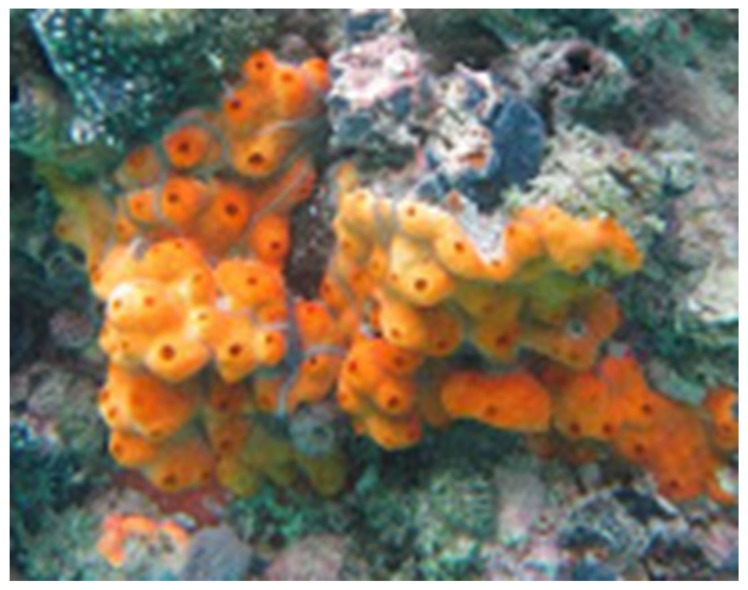
Underwater photograph of the Red Sea tunicate *Didemnum* species.

The marine tunicate *Didemnum* species (Figure 10) was harvested from Yanbu at the Saudi Red Sea coast by SCUBA diving at depths ranging between 2 and 7 m in May 2018. The tunicate was recognized by Dr. Shirley Parker-Nance at the Centre for African Conservation Ecology. The tunicate *Didemnum* species belongs to the Kingdom: Animalia, Phylum: Chordata, Subphylum: Tunicata, Class: Ascidiacea, Order: Aplousobranchia, and Family: Didemnidae. A voucher specimen under the code number DY-66 was deposited at the Red Sea Marine Invertebrates Collection at King Abdulaziz University.

### 3.3. Purification of Compounds ***1***–***7***

The freeze-dried tunicate (180 g) was macerated in a mixture of CH_2_Cl_2_-CH_3_OH (1:1) (3 × 300 mL) at room temperature. The extract was dried under reduced pressure to give a dried extract. The dried extract (5.32 g) was fractionated by VLC on the SiO_2_ column using hexane-CHCl_3_-MeOH gradients to afford seven main subfractions (A–G). The antiproliferative fraction E (1.2 g, IC_50_ = 15.2 µg/mL against PC-3), eluted with CHCl_3_-MeOH (9:1-8:2), was further fractionated on reversed-phase C18 VLC. The column was eluted with H_2_O/MeOH gradients. Proper fractions were assembled and dried under reduced pressure to afford eleven main subfractions (E-1 to E-11). The antiproliferative fraction E-1 (120 mg, IC_50_ = 11.5 µg/mL against PC-3) was subjected to reversed-phase HPLC (C_18_ Cosmosil ARII, 250 × 10 mm, 5 μm, Waters) started isocratically with 2% CH_3_CN in H_2_O for 10 min followed by a gradient system from 2% CH_3_CN to 20% CH_3_CN over 50 min to yield compounds **1** (*t*_R_ = 25.16 min, 4.3 mg), **2** (*t*_R_ = 27.48 min, 1.9 mg), **3** (*t*_R_ = 31.13 min, 4.2 mg), **4** (*t*_R_ = 13.70 min, 4.2 mg), **5** (*t*_R_ = 21.95 min, 3.4 mg), **6** (*t*_R_ = 7.33 min, 16.6 mg), and **7** (*t*_R_ = 6.20 min, 65 mg) (Figure S24).

### 3.4. Spectral Data of the Compounds

#### 3.4.1. Compound **1**

Faint Yellow Oil; [α]_D_ −55.6° (c 0.1, MeOH); (+)-HRESIMS *m*/*z* 319.0766 (Cald for C_10_H_13_N_6_O_5_ [M + Na]^+^, 319.0761); NMR Data: see Table 1.

#### 3.4.2. Compound **2**

Faint Yellow Oil; [α]_D_ −26.7° (c 0.1, MeOH); (+)-HRESIMS *m*/*z* 353.0738 (Calcd for C_10_H_15_N_6_O_5_P [M + Na]^+^, 353.0733); NMR Data: see Table 2.

#### 3.4.3. Compound **3**

White Powder; (+)-HRESIMS *m*/*z* 243.0127 (Calcd for C_8_H_4_N_4_O_4_ [M + Na]^+^, 243.0124); NMR Data: see Table 3.

#### 3.4.4. Compound **7**

Faint Yellowish Powder; (+)-LRESIMS *m*/*z* 60.0 [M + H]^+^; NMR Data: see Table 4.

### 3.5. Biological Evaluation of the Compounds

#### 3.5.1. Evaluation of the Antiproliferation Effects of the Compounds

The antiproliferative activity of the compounds was evaluated against colorectal cancer (SW-1222), breast cancer (MCF-7), and prostate cancer (PC-3). The cell lines utilized in this study were generously supplied by the Laboratory for Nanomedicine at King Abdullah University of Science and Technology (KAUST), KSA. Based on available records, these cell lines were initially sourced from a commercial supplier and have been regularly cultured and maintained within the KAUST facility. The antiproliferative effects were carried out using the MTT assay following established protocols [45,46,47]. Briefly, cells were seeded and allowed to adhere overnight in a humidified incubator at 37 °C with 5% CO_2_. Test compounds were initially added to the first row of a 96-well plate and subjected to 1:4 serial dilutions across the plate. Following treatment, cells were incubated for 72 h. Cell viability was assessed at 490 nm using the CellTiter 96^®^ AQueous Non-Radioactive Cell Proliferation Assay, and absorbance was measured with a Molecular Devices Emax microplate reader. IC_50_ values (μM) were calculated using SoftMax^®^ Pro software (Version 7.1.2) and are reported as the mean of three independent experiments. Data are from three individual experiments, each performed in triplicate, and presented as the IC_50_ ± SEM.

#### 3.5.2. Evaluation of the Antimicrobial Effects of the Compounds

Using well diffusion assay [48,49,50,51], the antimicrobial activities of compounds **1**–**7** were evaluated at 50 μg/well against various pathogenic microbes including methicillin-resistant *S. aureus* (MRSA) (ATCC^®^ 33591), *S. aureus* (ATCC^®^ 12600), *P. aeruginosa* (ATCC^®^ 9027), and *E. coli* (ATCC^®^ 11775).

### 3.6. Molecular Docking on Potential Targets for Antiproliferative Activity

To rationalize the antiproliferative activity of the top three active compounds against each cell line, molecular target predictions were conducted using the SwissTargetPrediction (http://www.swisstargetprediction.ch/, accessed on 22 March 2025), PharmMapper (http://www.lilab-ecust.cn/pharmmapper/, accessed on 22 March 2025), and SEA (https://sea.bkslab.org/, accessed on 22 March 2025) databases. Additionally, the GeneCards database (https://www.genecards.org/, accessed on 22 March 2025) was employed to identify genes associated with human breast, colon, and prostate cancers. To investigate the molecular basis for the anti-cancer activity of these compounds, Venny2.1.0 BioinfoGP (https://bioinfogp.cnb.csic.es/tools/venny/, accessed on 22 March 2025) was used to find common targets shared between human breast, colon, and prostate cancers, and the active compounds.

Molecular docking studies of the three most active inhibitors for each cell line were performed on estrogen receptor alpha (ESR1), human topoisomerase II alpha (TOP2A), and cyclin-dependent kinase 5 (CDK5). The top three active compounds were imported into the LigPrep wizard of Maestro 10.2 (Schrödinger) software as 3D structures in SDF format for energy minimization. Ionization at pH 7 was performed to generate all possible states of the compounds. High-resolution crystal structures of the target proteins were obtained from the Protein Data Bank (PDB). The ESR1 structure co-crystallized with estradiol as the native ligand (PDB ID 6CBZ) was used, while the TOP2A structure with etoposide as the co-crystallized inhibitor (PDB ID 5GWK) was selected. The crystal structure of CDK5 co-crystallized with indirubin-3′-monoxime (PDB ID 1UNH) was also chosen.

Protein preparation was carried out using the Protein Preparation Wizard in Maestro, where hydrogen atoms and bond orders were assigned, and water molecules more than 5 Å away from the active site were removed. Protonation at pH 7 was performed using PROPKA, followed by energy minimization using the OPLS 3 force field until the root mean square deviation (RMSD) of the minimized structure relative to the crystal structure was less than 0.3 Å. Receptor grid generation was performed using a box centered around the centroid of the co-crystallized ligand. Finally, molecular docking was carried out using Glide in Maestro, employing the extra-precision (XP) mode.

## 4. Conclusions

The present study highlights the untapped potential of marine tunicates, specifically the Red Sea tunicate *Didemnum* species, as a rich source of bioactive secondary metabolites with promising anticancer properties. Among the isolated compounds, didemnosides A and B (**1** and **2**) exhibited notable cytotoxicity against cancer cell lines, with compound **1** demonstrating the strongest inhibitory effects on MCF-7 cells. The molecular docking studies further provided insights into the compounds’ interactions with critical cancer-related targets, including estrogen receptor alpha (ESR1), human topoisomerase II alpha (TOP2A), and cyclin-dependent kinase 5 (CDK5), suggesting that these compounds may be valuable in cancer therapy.

The findings underscore the potential of marine-derived nucleosides as scaffolds for the development of novel anticancer agents through derivatization. While compound **1** shows considerable promise, future studies should focus on optimizing its structure to improve potency and selectivity. Additionally, exploring the synergistic effects of these compounds in combination with other anticancer agents may enhance therapeutic efficacy.

In terms of future trends, there is a growing emphasis on the discovery of natural products from marine sources as leading to drug development. The application of advanced techniques like molecular docking and high-throughput screening will facilitate the identification of new drug candidates. Moreover, integrating marine natural product research with computational drug design and medicinal chemistry holds great promise for the discovery of more effective and targeted cancer therapies. The future direction should also include in vivo studies and clinical trials to evaluate the safety, pharmacokinetics, and therapeutic potential of these compounds, paving the way for their eventual clinical application in oncology.

## Figures and Tables

**Table 1:** NMR data of compound **1** (600 MHz for ^1^H and 150 MHz for ^13^C, DMSO-*d*_6_).

Position	δ_C_, Type	δ_H_ [Mult., *J* (Hz)]	HMBC	ROESY
2	145.9, CH	8.05 (s)	C-4	H_2_-5′
4	148.2, C			
5	124.4, C			
6	156.6, C			
8	138.7, CH	8.31 (s)	C-4, C-5, C-1′	H-1′
1′	87.4, CH	5.85 (d, 5.7)	C-4, C-8, C-3′, C-4′	H-8, H-4′
2′	74.1, CH	4.46 (t, 5.2)	C-1′, C-3′, C-4’	H-3’
O*H*-2’		5.52 (brs) ^a^		
3’	70.3, CH	4.11 (dd, 4.6, 3.8)	C-1’, C-4’, C-5’	H-2’, H_2_-5’
O*H*-3’		5.25 (brs) ^a^		
4’	85.6, CH	3.92 (q, 3.8)	C-2’, C-3’, C-5’	H-1’
5’	61.3, CH_2_	3.64 (dd, 12.0, 4.0) 3.54 (dd, 12.0, 4.0)	C-2’, C-3’, C-4’	H-2, H-3’
O*H*-5’		5.08 (brs) ^a^		

^a^ Interchangeable signals.

**Table 2:** NMR data of compound **2** (600 MHz for ^1^H and 150 MHz for ^13^C, DMSO-*d*_6_).

Position	δ_C_, Type	δ_H_ [Mult., *J* (Hz)]	HMBC	ROESY
2	146.2, CH	8.04 (s)	C-4, C-5, C-6	H_2_-5′
4	147.9, C			
5	124.4, C			
6	157.1, C			
8	138.3, CH	8.27 (s)	C-4, C-5, C-1′	H-1′
1′	83.6, CH	6.30 (dd, 7.2, 6.3)	C-4, C-8, C-3′, C-4′, C-5′	H-8, H-4′
2′	39.9, CH_2_	2.63 (ddd, 13.3, 7.5, 5.8) 2.28 (ddd, 13.3, 6.2, 3.2)	C-1′, C-3′, C-4′	H-3′
3′	70.6, CH	4.38 (quin., 3.0)	C-1′, C-2′, C-4′, C-5′	H_2_-2′, H_2_-5′
O*H*-3′		5.34 (brs) ^a^		
4′	87.9, CH	3.86 (q, 4.4)	C-1′, C-2′, C-3′, C-5′	H-1′
5′	61.6, CH_2_	3.59 (dd, 11.8, 4.6) 3.51 (dd, 11.8, 4.4)	C-1′, C-3′, C-4′	H-2, H-3′
O*H*-5‘		5.08 (brs) ^a^		

^a^ Interchangeable signals.

**Table 3:** NMR data of compound **3** (600 MHz for ^1^H and 150 MHz for ^13^C, DMSO-*d*_6_).

Position	δ_C_, Type	δ_H_ [Mult., *J* (Hz)]
2,2′	151.5, C	
4,4′	164.3, C	
5,5′	100.2, CH	5.45 (d, 7.6)
6,6′	142.2, CH	7.39 (d, 7.6)

**Table 4:** NMR data of compound **7** (600 MHz for ^1^H and 150 MHz for ^13^C, DMSO-*d_6_*).

Position	δ_C_, Type	δ_H_ [Mult., *J* (Hz)]
1	22.5, CH_3_	1.75 (s)
2	171.6, C	
NH_2_		7.40 (brs) 6.68 (brs)

**Table 5:** Antiproliferative effects of compounds **1**–**7**.

Compound	Average IC_50_ (μM) ± SEM		
	MCF-7	SW1222	PC-3
**1**	0.597 ± 0.05	6.696 ± 0.69	9.365 ± 1.02
**2**	≥50	6.392 ± 0.62	7.215 ± 0.81
**3**	NT	NT	NT
**4**	≥50	7.072 ± 0.73	9.686 ± 0.98
**5**	≥50	5.254 ± 0.56	7.167 ± 0.75
**6**	≥50	6.191 ± 0.69	8.163 ± 0.91
**7**	6.574 ± 0.57	5.649 ± 0.58	7.478 ± 0.76
Doxorubicin ^a^	0.4477 ± 0.04	0.4364 ± 0.038	2.627 ± 0.25

^a^ Positive anticancer drug; NT = not tested.

**Table 6:** Docking XP Gscores in Kcal mol^−1^ of top antiproliferative compounds with ESR1 (PDB ID: 6CBZ), TOP2A (PDB ID: 5GWK), and CDK5 (PDB ID: 1UNH).

Compound	XP Gscore		
	ESR1 (6CBZ)	TOP2A (5GWK)	CDK5 (1UNH)
**1**	−7.700	≥−5.000	≥−5.000
**2**	≥−5.000	−6.281	−7.649
**4**	≥−5.000	≥−5.000	−8.907
**6**	≥−5.000	≥−5.000	≥−5.000
**7**	≥−5.000	≥−5.000	≥−5.000

## Data Availability

The original contributions presented in this study are included in the article and Supplementary Materials.

## Supplementary Materials

The following are available online at https://www.mdpi.com/article/10.3390/md23070262/s1, Figures S1–S20: (+)-HRESIMS of compounds **1**–**3**, (+)-LRESIMS of compound **4**, One- and two-dimensional NMR spectra of didemnoside A (**1**), didemnoside B (**2**), 1,1′,3,3′-bisuracil (**3**) and acetamide (**7**). Figure S21: 3D and 2D interaction diagrams of estradiol with the crystal structure of ESR1 (PDB ID: 6CBZ). Figure S22: **3**D and **2**D interaction diagrams of etoposide with the crystal structure of TOP2A (PDB ID: 5GWK). Figure S23: 3D and 2D interaction diagrams of indirubin-3′-monoxime with the crystal structure of CDK5 (PDB ID: 1UNH). Figure S24: HPLC chromatogram of purification of compounds **1**–**7**.
