# Supplementary material for: Didemnosides A and B: Antiproliferative Nucleosides from the Red Sea Marine Tunicate Didemnum Species
Source: Mar Drugs. 2025 Jun 23;23(7):262. doi: 10.3390/md23070262 (PMC12298903; doi:10.3390/md23070262)

Supplementary Information

	Figure
(+)-HRESIMS spectrum of compound **1**	**S1**
^{1}H NMR spectrum of compound **1** (DMSO-d_6)	**S2**
^{13}C NMR spectrum of compound **1** (DMSO-d_6)	**S3**
^{1}H-^{1}H COSY spectrum of compound **1** (DMSO-d_6)	**S4**
HSQC spectrum of compound **1** (DMSO-d_6)	**S5**
HMBC spectrum of Compound **1** (DMSO-d_6)	**S6**
^{1}H-^{1}H ROESY spectrum of compound **1** (DMSO-d_6)	**S7**
(+)-HRESIMS spectrum of compound **2**	**S8**
^{1}H NMR spectrum of compound **2** (DMSO-d_6)	**S9**
^{13}C NMR spectrum of compound **2** (DMSO-d_6)	**S10**
^{1}H-^{1}H COSY spectrum of compound **2** (DMSO-d_6)	**S11**
HSQC spectrum of compound **2** (DMSO-d_6)	**S12**
HMBC spectrum of compound **2** (DMSO-d_6)	**S13**
^{1}H-^{1}H ROESY spectrum of compound **2** (DMSO-d_6)	**S14**
(+)-HRESIMS spectrum of compound **3**	**S15**
^{1}H NMR spectrum of compound **3** (DMSO-d_6)	**S16**
^{13}C NMR spectrum of compound **3** (DMSO-d_6)	**S17**
(+)-LRESIMS spectrum of compound **7**	**S18**
^{1}H NMR spectrum of compound **7** (DMSO-d_6)	**S19**
^{13}C NMR spectrum of compound **7** (DMSO-d_6)	**S20**
3D (left) and 2D (right) interaction diagrams of estradiol with the crystal structure of ESR1 (PDB ID: 6CBZ)	**S21**
3D (left) and 2D (right) interaction diagrams of etoposide with the crystal structure of TOP2A (PDB ID: 5GWK)	**S22**
3D (left) and 2D (right) interaction diagrams of indirubin-3'-monoxime with the crystal structure of CDK5 (PDB ID: 1UNH)	**S23**
HPLC chromatogram of purification of compounds **1-7**	**S24**

Figure S1. (+)-HRESIMS spectrum of compound **1**.

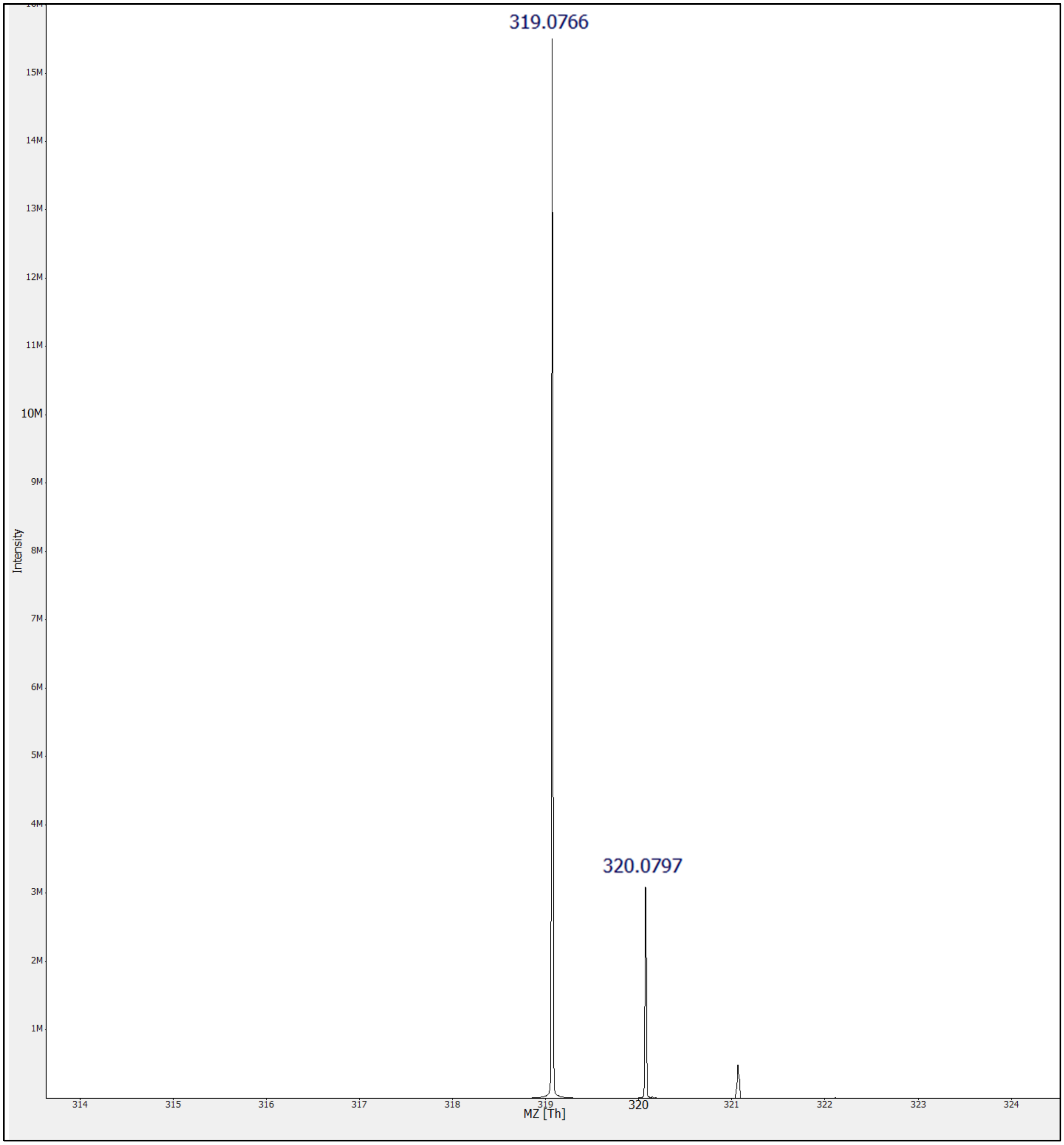

Figure S2. ^{1}H NMR spectrum of compound **1** (DMSO-d_6).

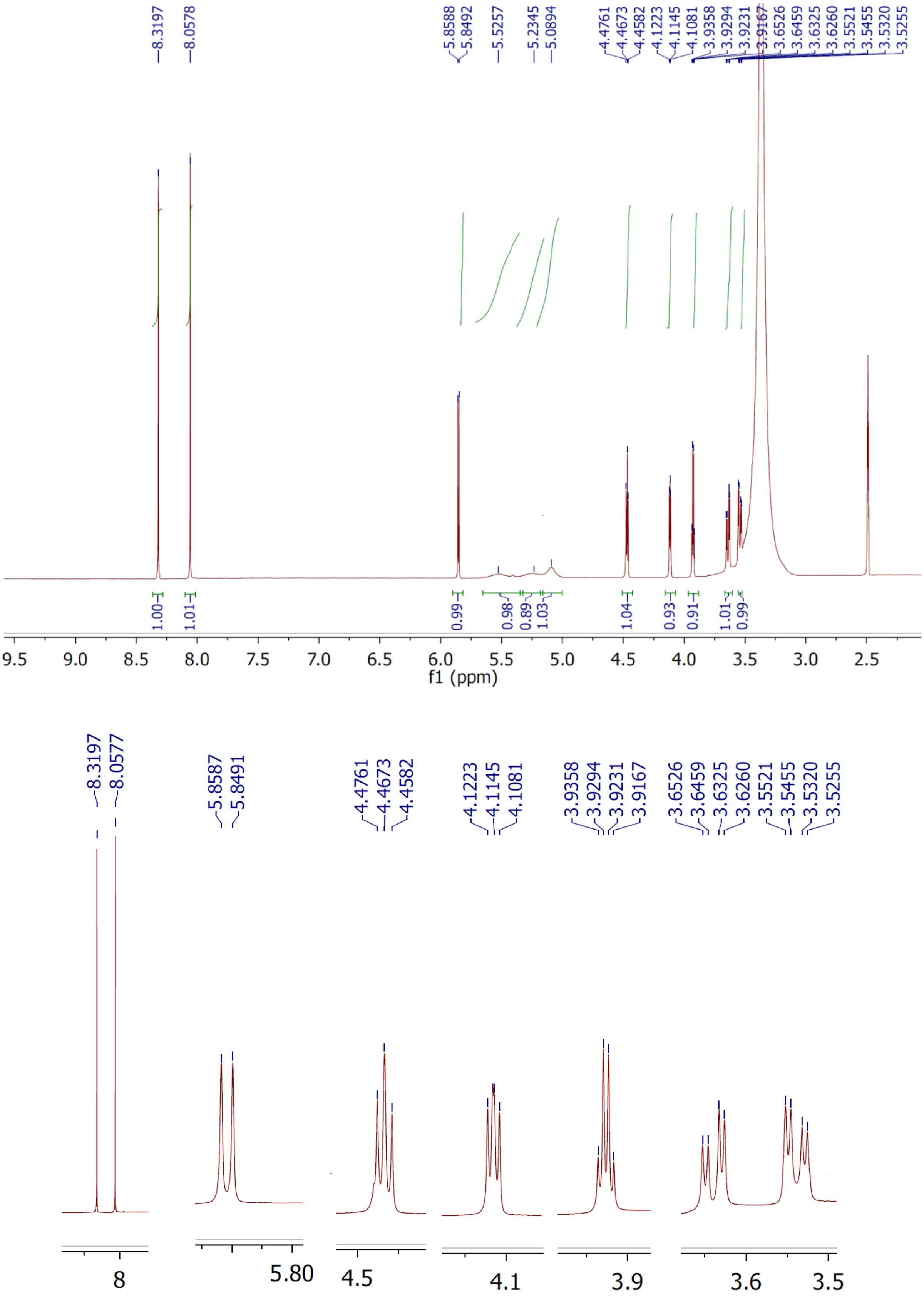

Figure S3. ^{13}C NMR spectrum of compound **1** (DMSO-*d*$_6$).

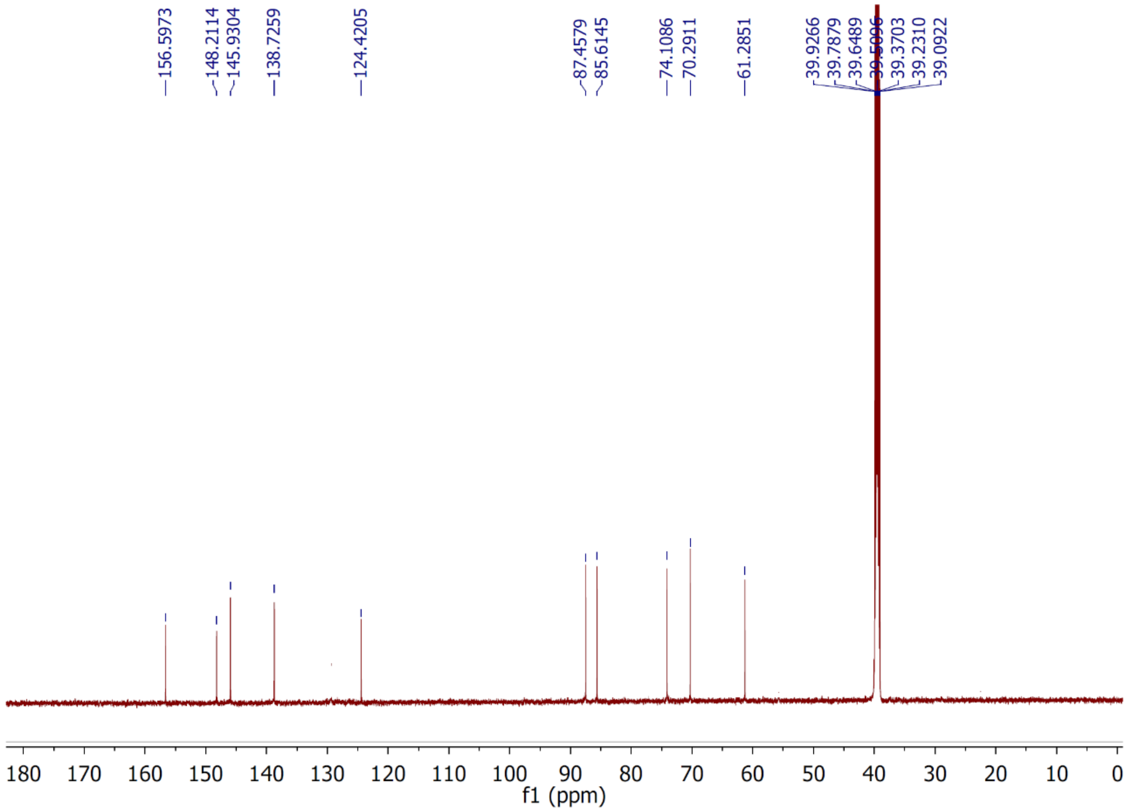

Figure S4. ^{1}H-^{1}H COSY spectrum of compound **1** (DMSO-*d*$_6$).

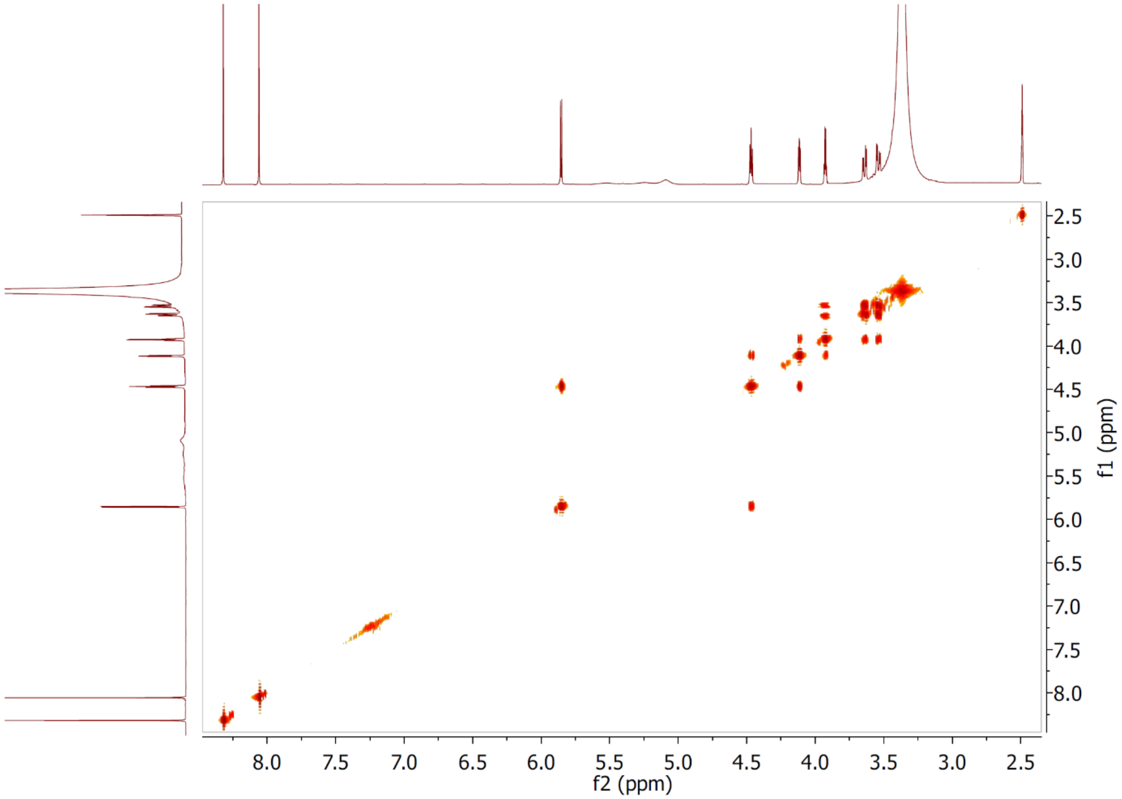

Figure S5. HSQC spectrum of compound **1** (DMSO-d_6).

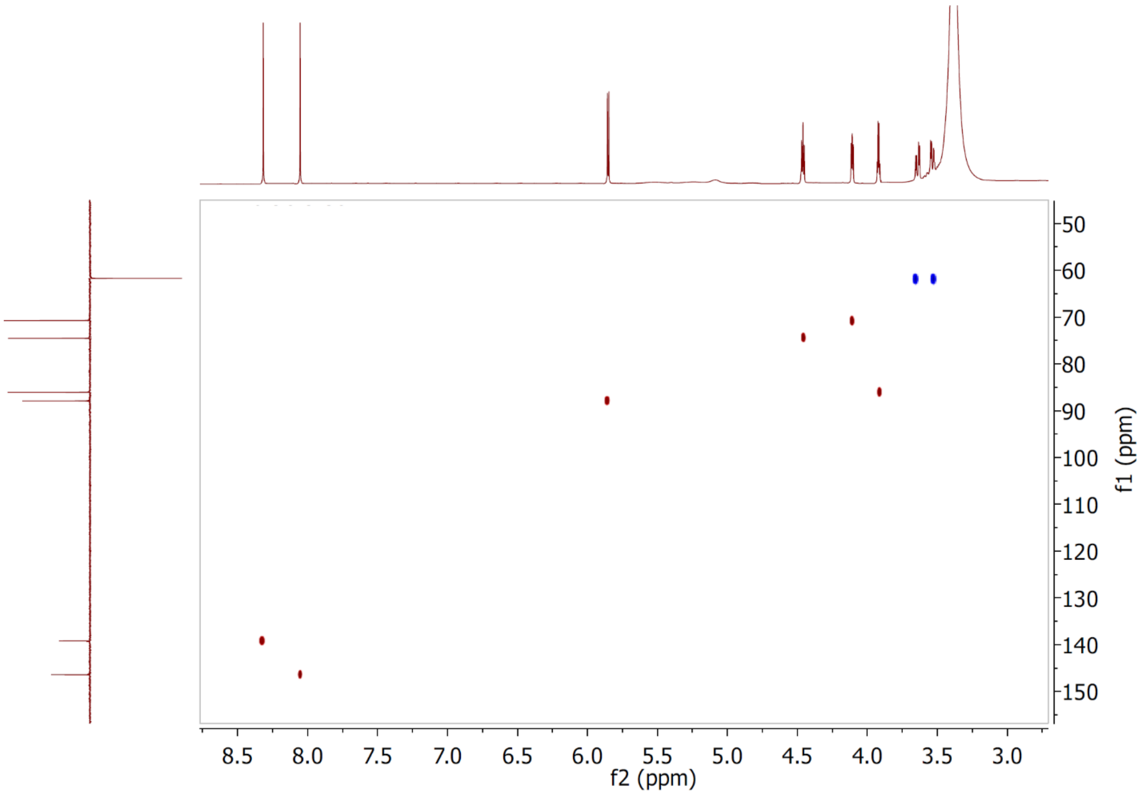

Figure S6. HMBC spectrum of compound **1** (DMSO-d_6).

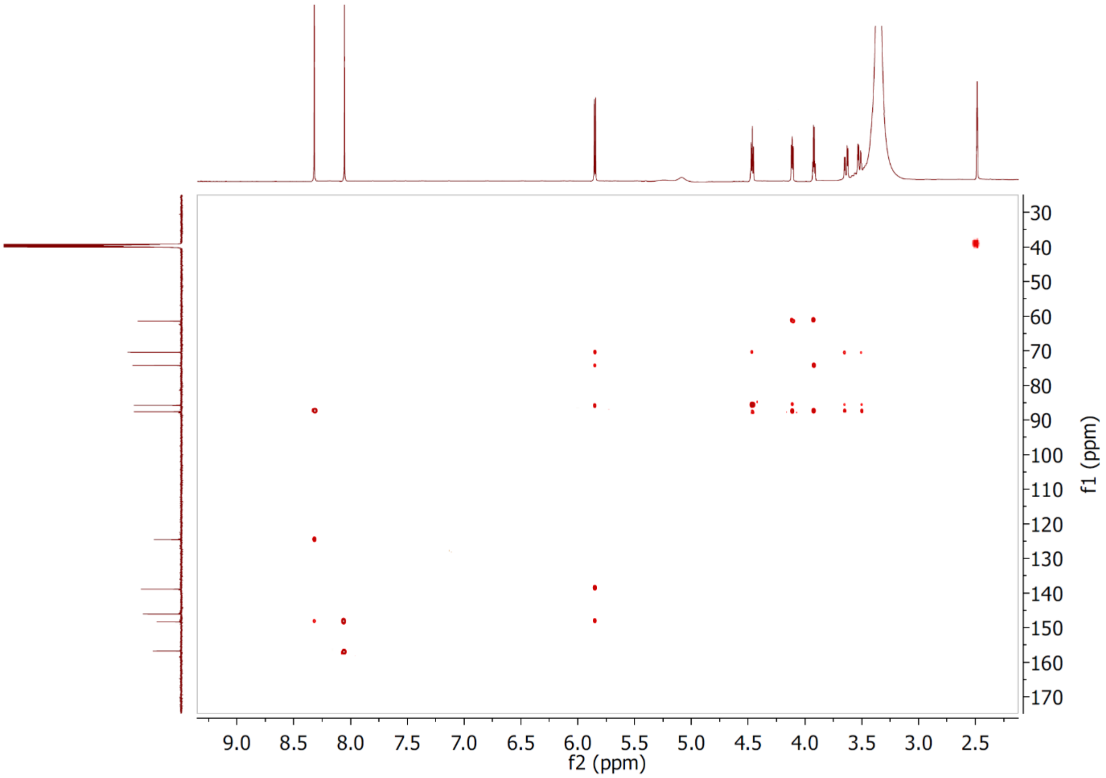

Figure S7. ^{1}H-^{1}H ROESY spectrum of compound **1** (DMSO-d_6).

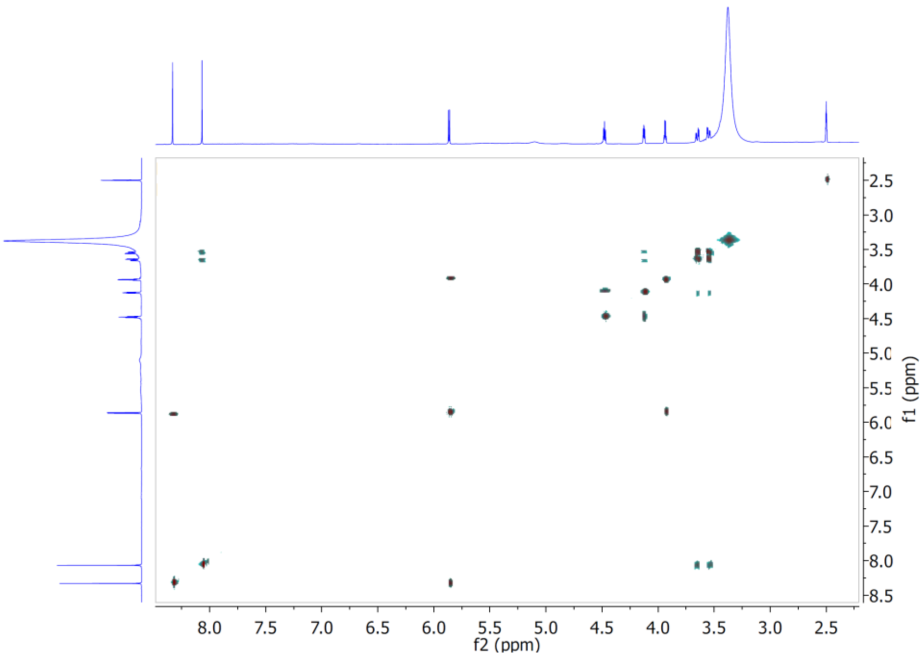

Figure S8. (+)-HRESIMS spectrum of compound **2**.

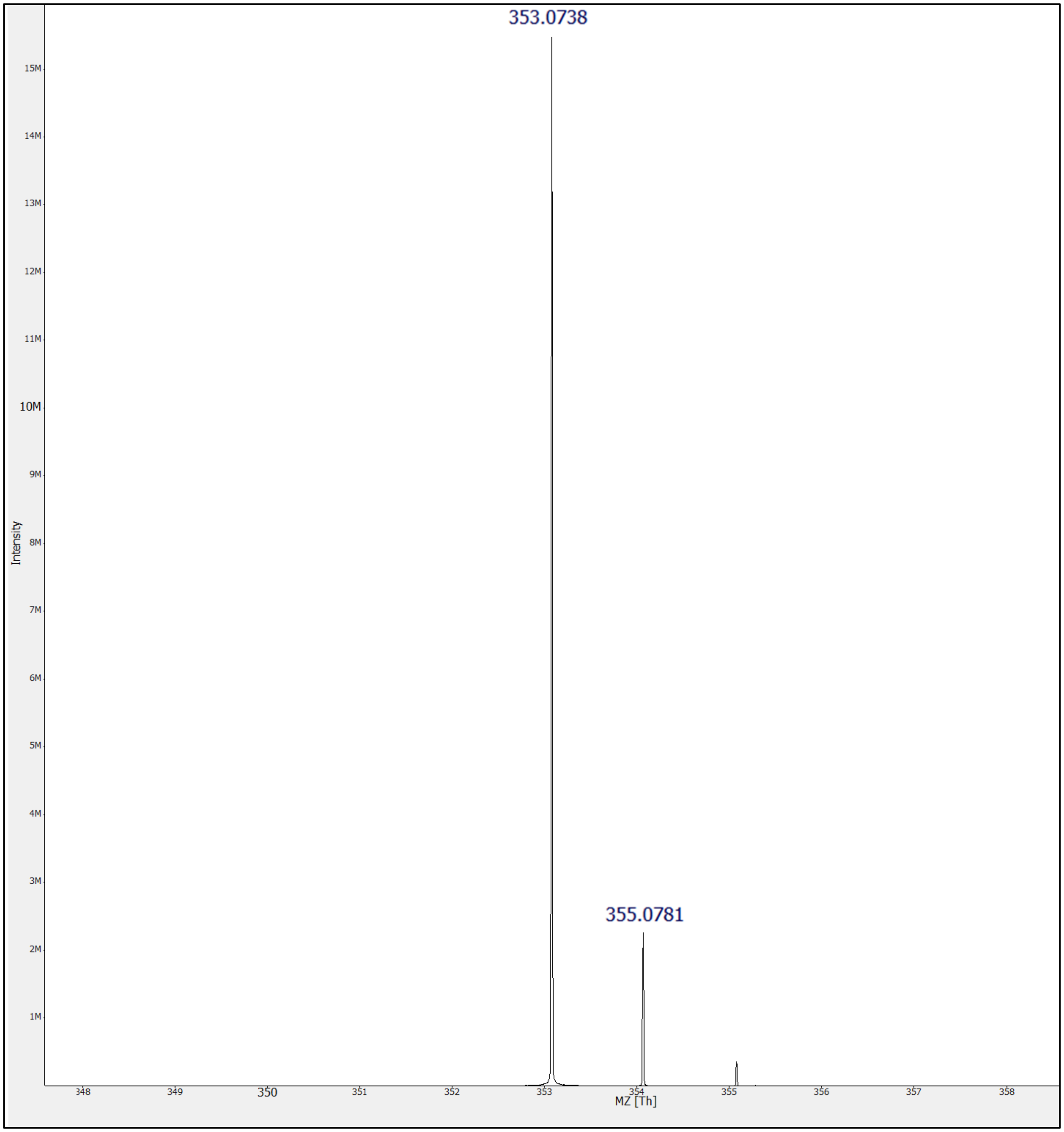

Figure S9. ^{1}H NMR spectrum of compound **2** (DMSO-d_6).

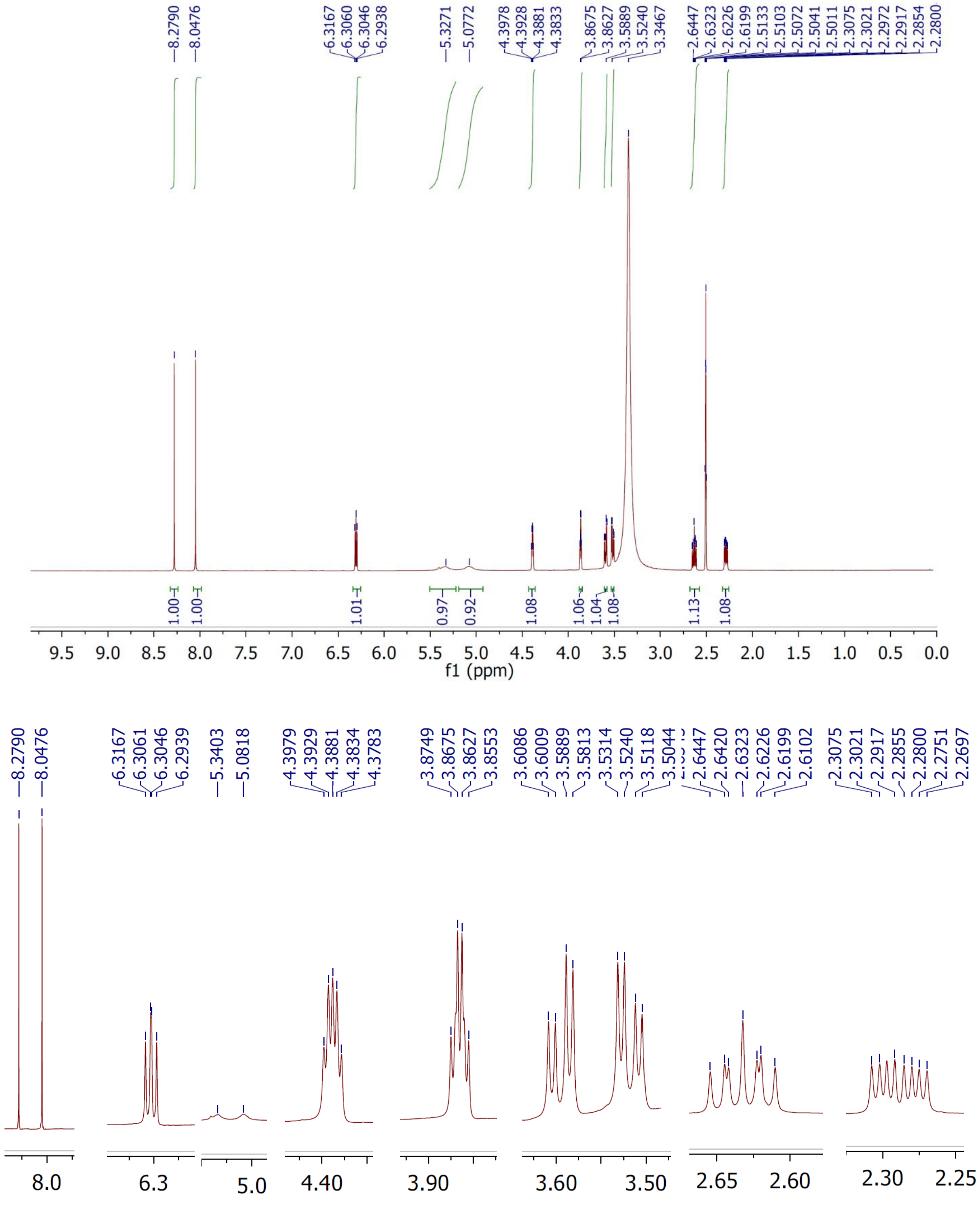

Figure S10. ^{13}C MMR spectrum of compound **2** (DMSO-d_6).

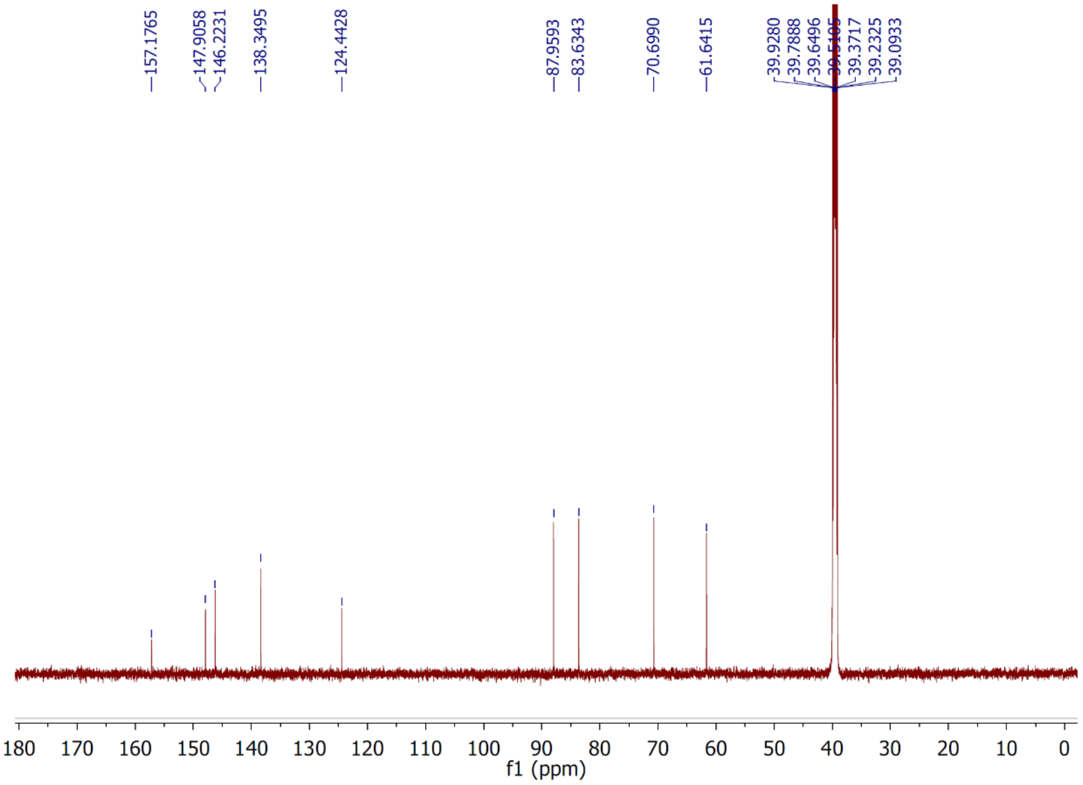

Figure S11. ^{1}H-^{1}H COSY spectrum of compound **2** (DMSO-d_6).

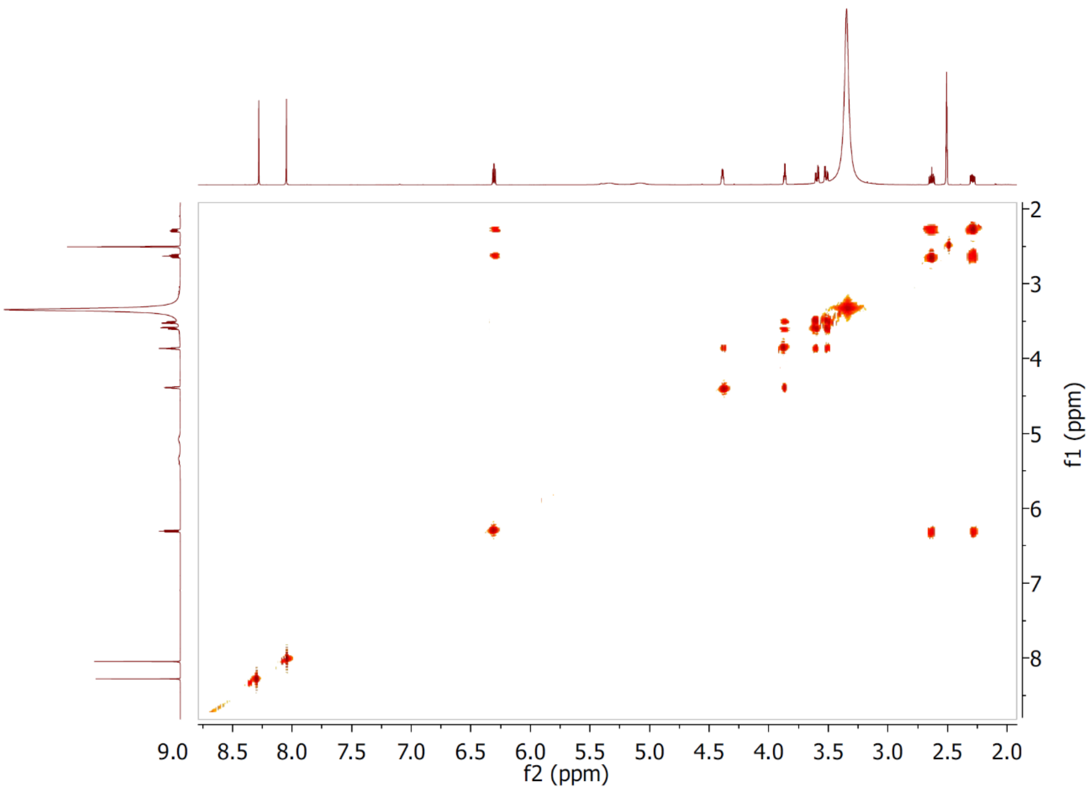

Figure S12. HSQC spectrum of compound **2** (DMSO-d_6).

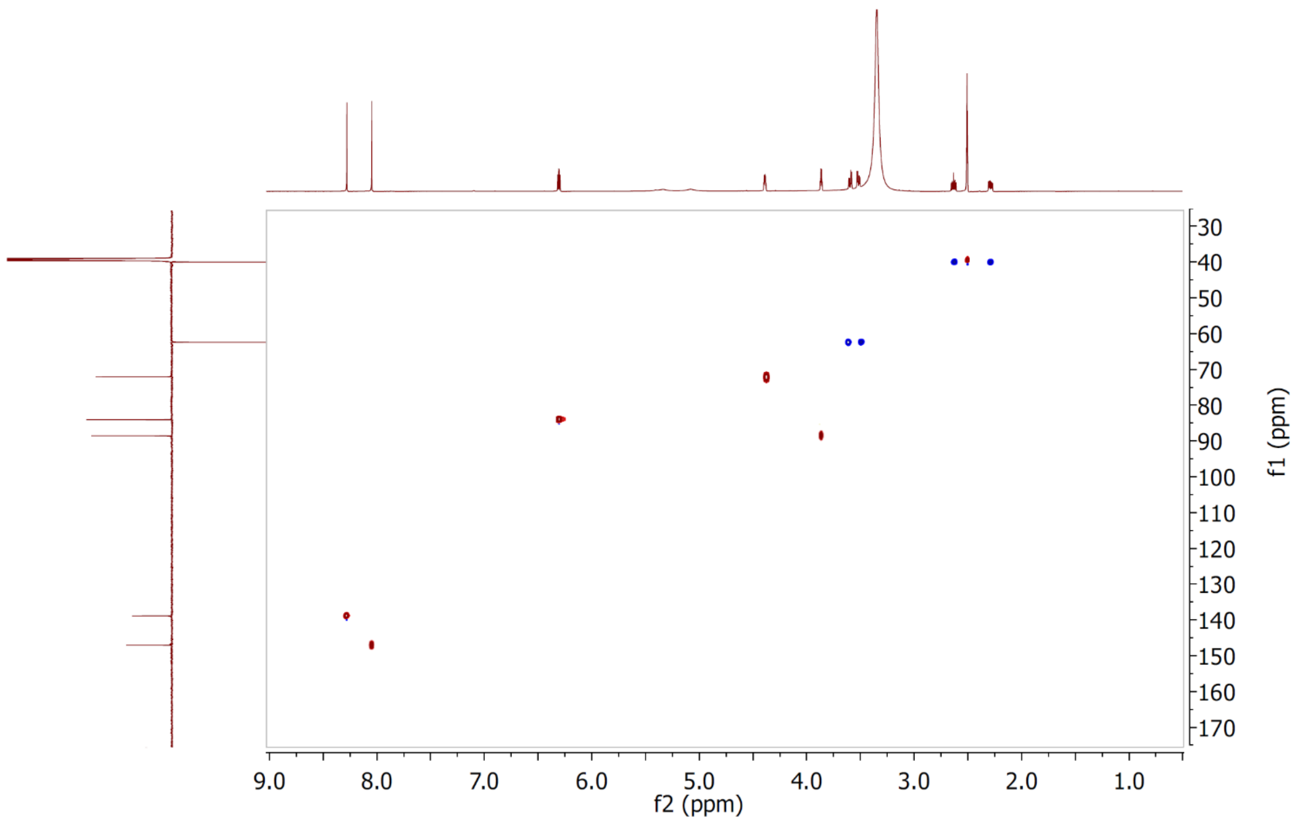

Figure S13. HMBC spectrum of compound **2** (DMSO-d_6).

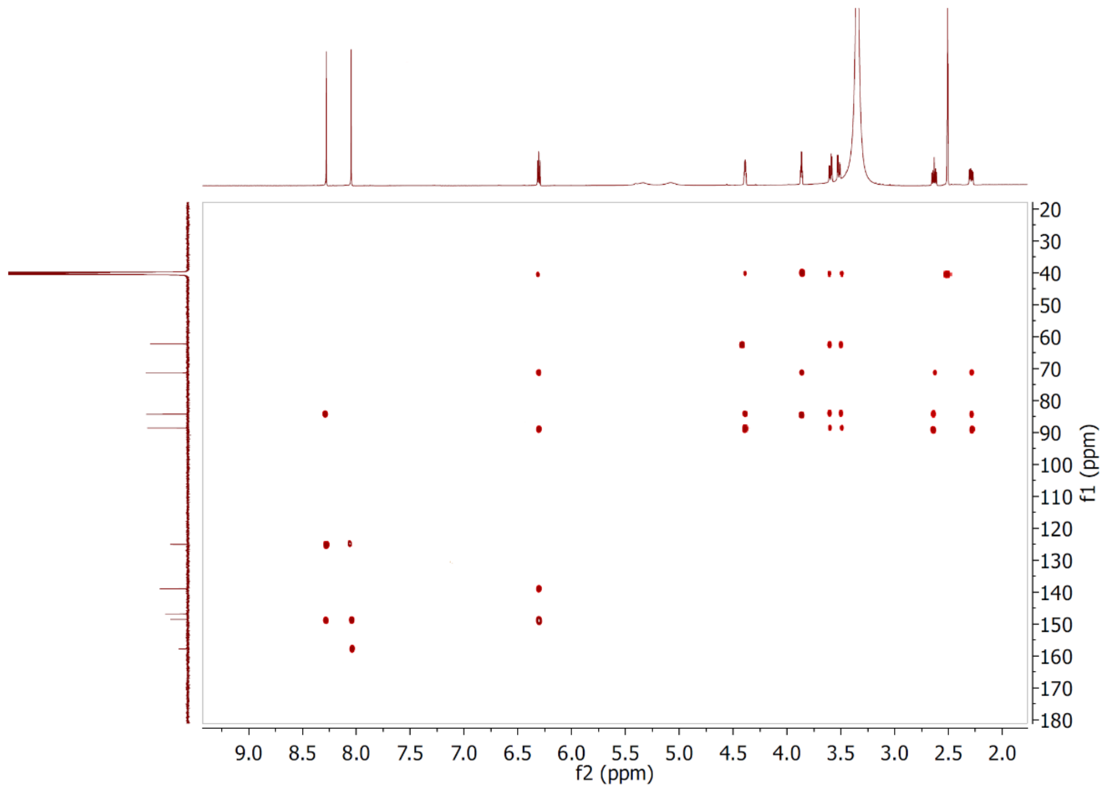

Figure S14. ^{1}H-^{1}H ROESY spectrum of compound **2** (DMSO-d_6).

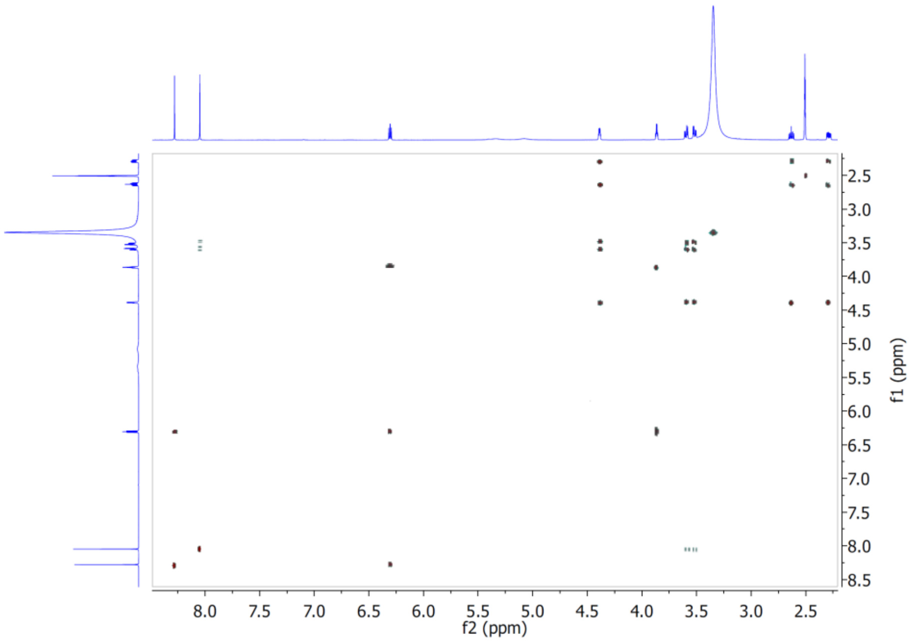

Figure S15. (+)-HRESIMS spectrum of compound **3**.

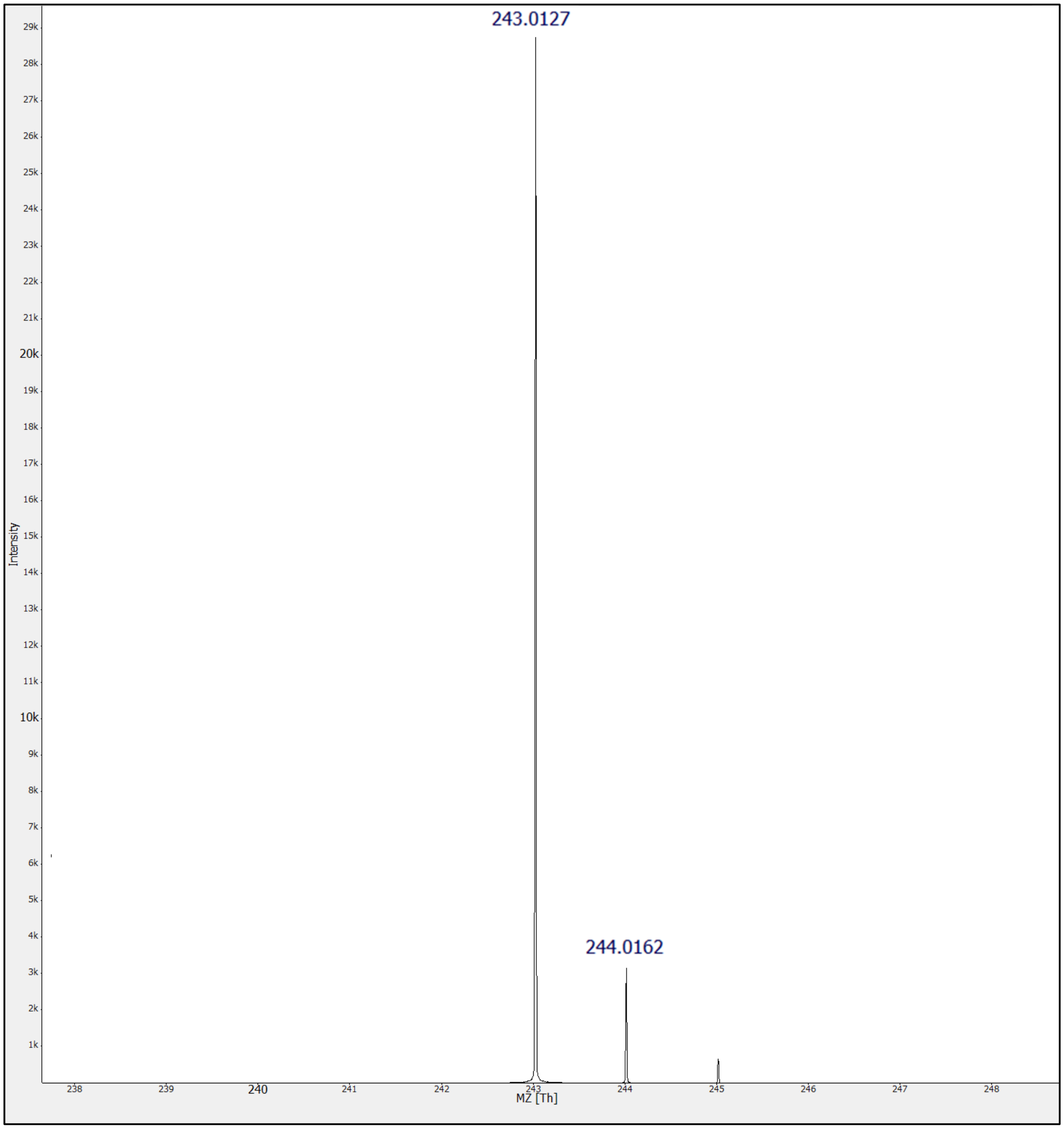

Figure S16. ^{1}H NMr spectrum of compound **3** (DMSO-d_6).

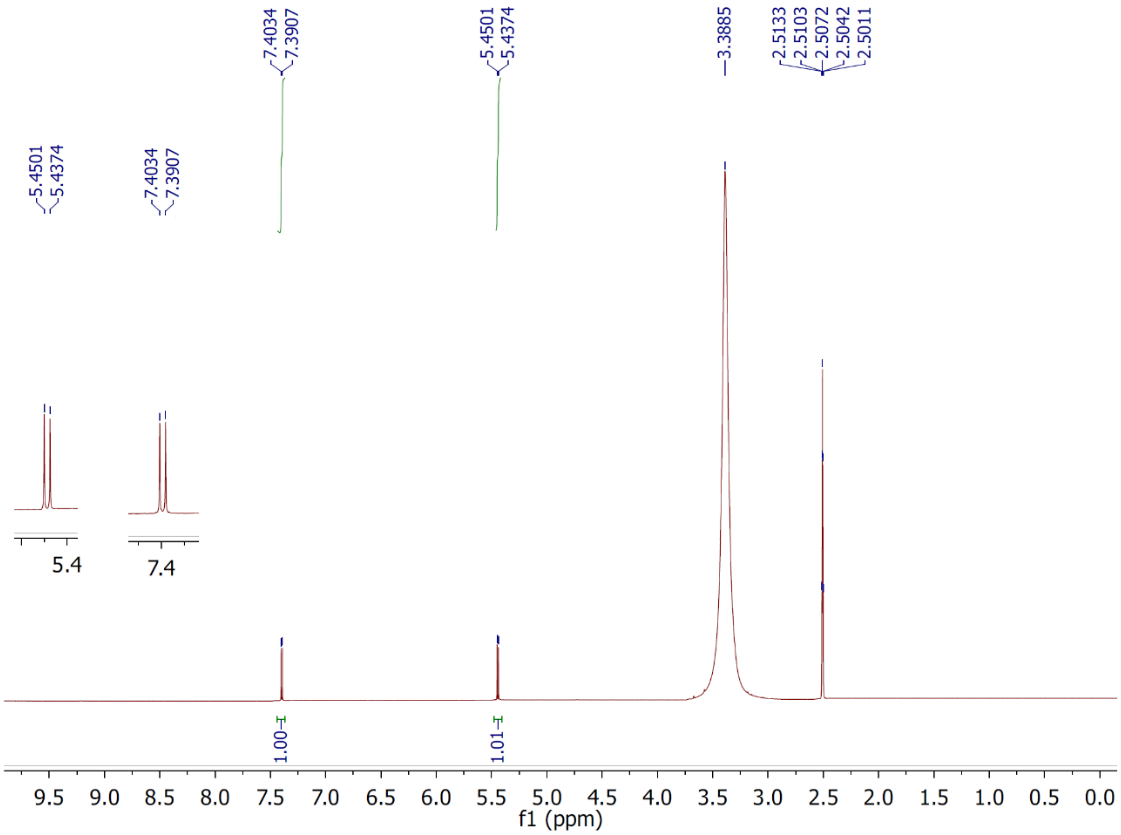

Figure S17. ^{13}C NMR spectrum of compound **3** (DMSO-d_6).

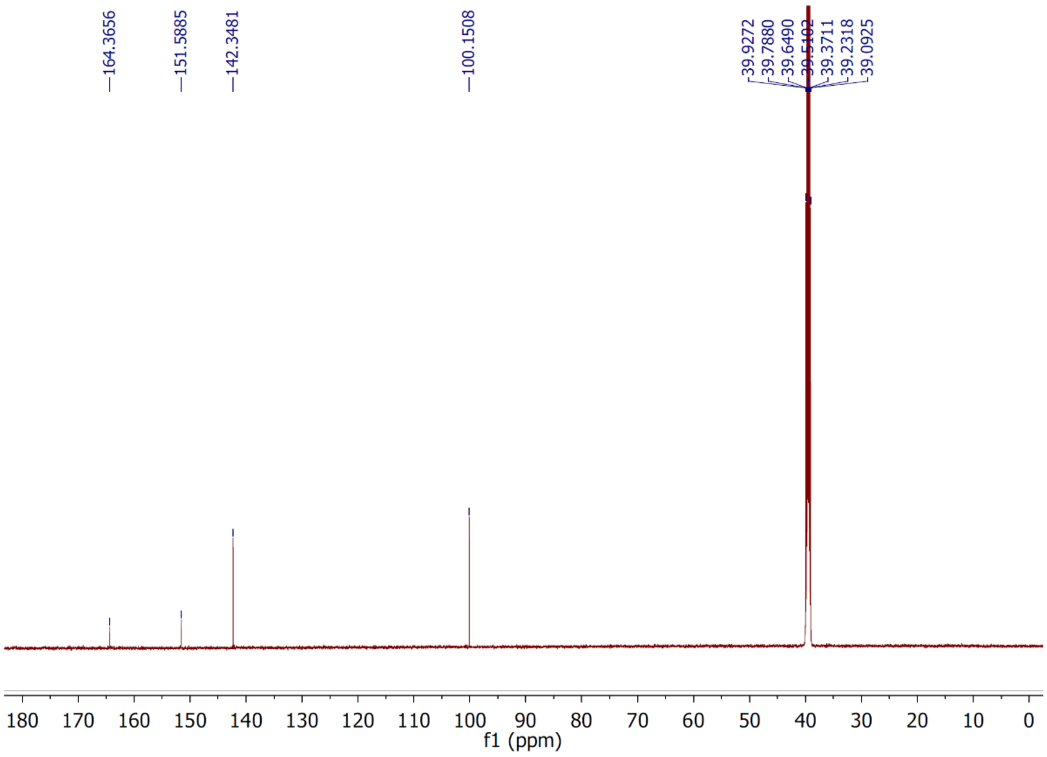

Figure S18. (+)-LRESIMS spectrum of compound **7**.

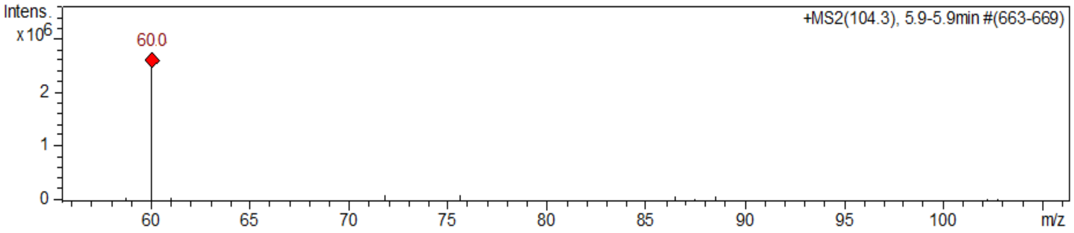

Figure S19. ^{1}H NMR spectrum of compound **7** (DMSO-d_6).

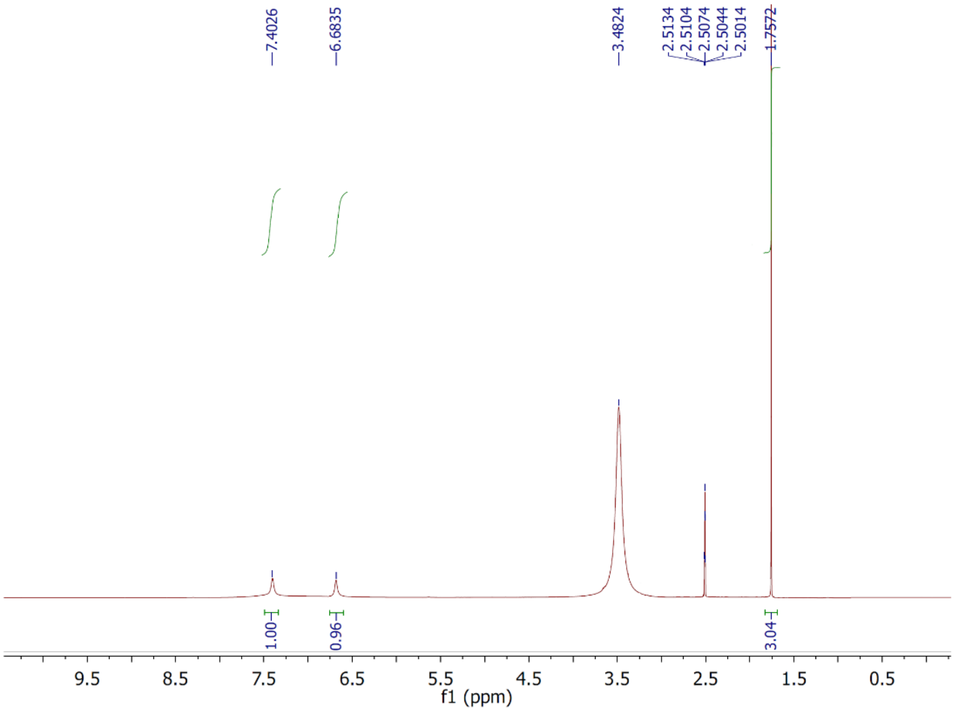

Figure S20. ^{13}C NMR spectrum of compound **7** (DMSO-d_6).

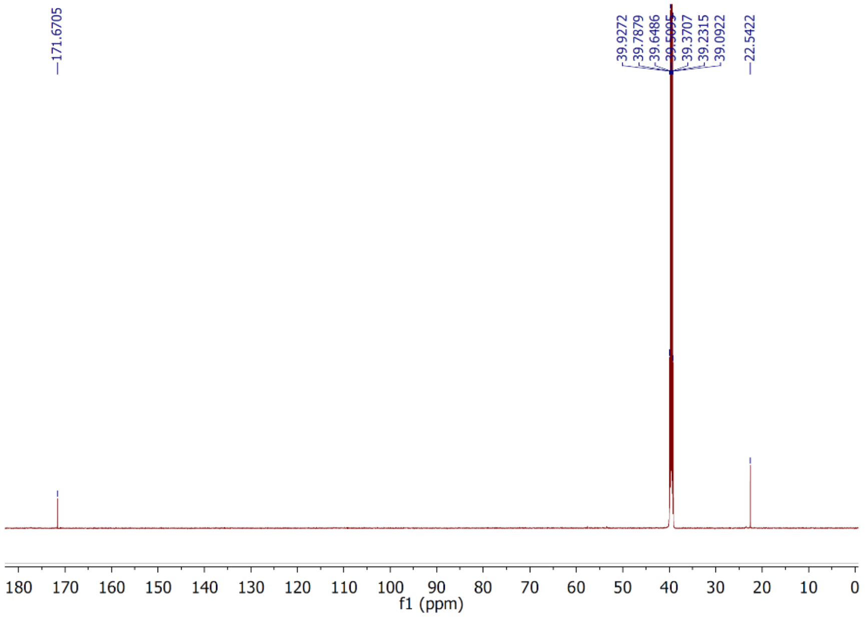

Figure S21. 3D (left) and 2D (right) interaction diagrams of estradiol with the crystal structure of ESR1 (PDB ID: 6CBZ).

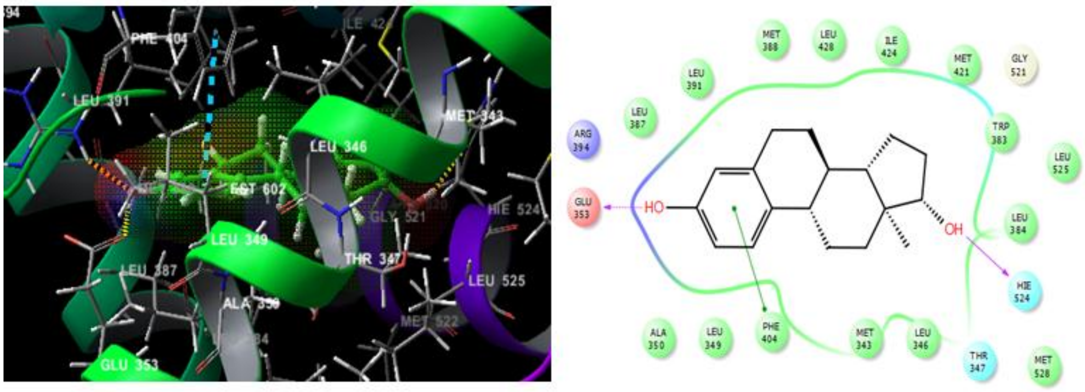

Figure S22. 3D (left) and 2D (right) interaction diagrams of etoposide with the crystal structure of TOP2A (PDB ID: 5GWK).

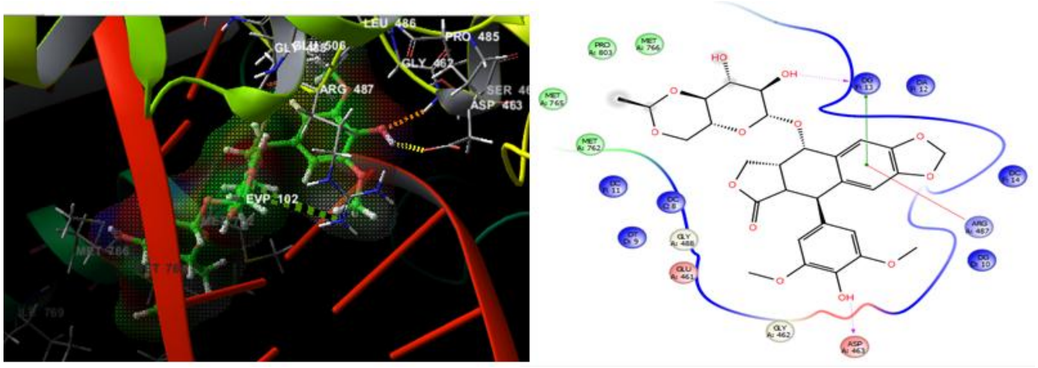

Figure S23. 3D (left) and 2D (right) interaction diagrams of indirubin-3'-monoxime with the crystal structure of CDK5 (PDB ID: 1UNH).

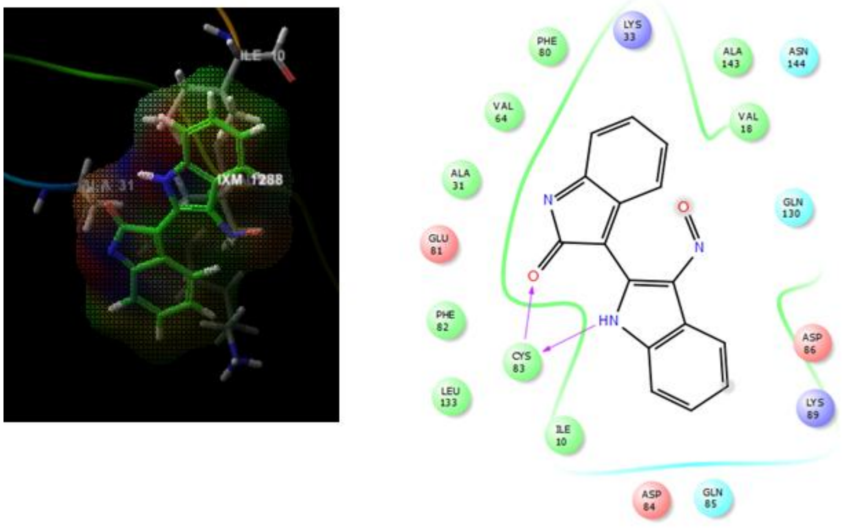

Figure S24. HPLC chromatogram of purification of compounds **1-7**.

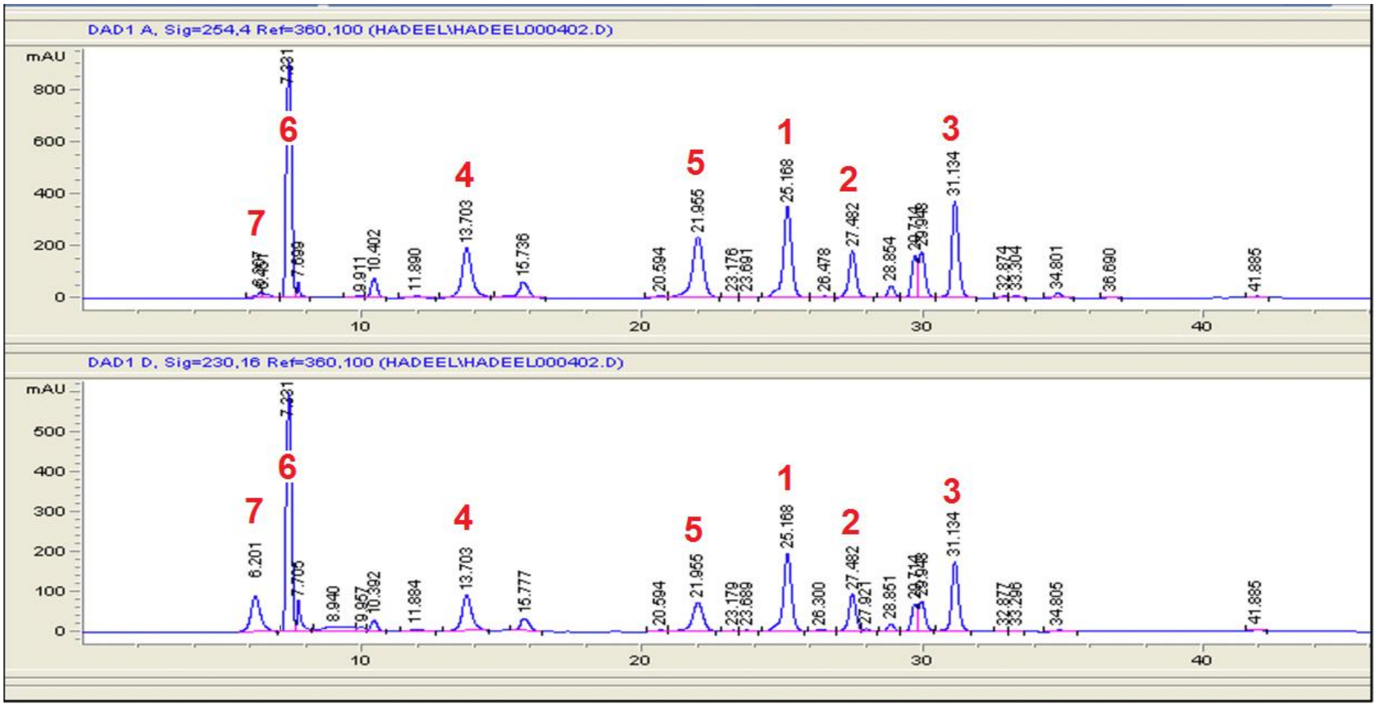

Supplement: Supplementary file 1 [file marinedrugs-23-00262-s001.zip › marinedrugs-3691066-supplementary.pdf]
